# Two-stage DRG grouping of cerebral infarction based on comorbidity and complications classification

**DOI:** 10.3389/fpubh.2025.1513744

**Published:** 2025-04-28

**Authors:** Siyu Zeng, Lele Li, Jialing Li, Xiaozhou He

**Affiliations:** ^1^School of Logistics, Chengdu University of Information Technology, Chengdu, Sichuan, China; ^2^School of Labor and Human Resources, Renmin University of China, Beijing, China; ^3^Institute for Hospital Management of Henan Province, Zhengzhou, China; ^4^School of Management, Hunan University of Technology and Business, Changsha, Hunan, China; ^5^Business School, Sichuan University, Chengdu, Sichuan, China

**Keywords:** diagnosis-related groups, classification, comorbidity and complications, cerebral infarction, two-stage grouping method

## Abstract

**Background:**

Since 2017, cerebral infarction (CI) has become a leading cause of mortality in China, with rising treatment costs posing significant challenges to the healthcare system. The Diagnosis-Related Groups (DRG) payment system has been recognized as a potential solution to curb rising healthcare expenditures. However, in its implementation, China faces considerable hurdles due to its vast geographical size, regional economic disparities, and heterogeneous disease spectrum.

**Objective:**

This study proposes a novel two-stage grouping strategy with a two-stage method tailored to address the local context of western China. The method adaptively accommodates regional variations in disease burden and healthcare resource distribution.

**Methods:**

Using hospitalization data from 111,025 CI patients collected by the Healthcare Security Administration of a western Chinese city between 2016 and 2018 (during the pre-DRG implementation period), we developed a two-stage DRG method. In the first stage, regression analysis identified and prioritized comorbidities and complications that influence medical costs. In the second stage, a decision tree algorithm established standardized classification protocols for DRG grouping, ensuring regional adaptability.

**Results:**

The average hospitalization cost for CI patients was USD$ 1,565, with total expenditures reaching USD$ 1.71 million in the target city. By employing this localized two-stage grouping model, the proportion of inter-group variations, as measured by the coefficient of variation (CV), is below 1, reaching 100%, satisfying the technical criteria for DRG categorization. This optimization reduced the number of DRG from 18 to 4. It increased the proportion of groups with CV to <0.8 from 67 to 100%, signifying a substantial enhancement in group heterogeneity compared to the existing grouping method, China Healthcare Security Diagnosis-Related Groups (CHS-DRG).

**Conclusion:**

This study demonstrates the effectiveness of our proposed two-stage method using real data. Implementation of this localized method in the target city could result in potential savings of USD$ 8.59 million, surpassing the existing CHS-DRG method. These findings suggest that this adaptive method may be a scalable strategy for resource-limited regions undergoing healthcare system reforms.

## 1 Introduction

Cerebrovascular disease is the leading cause of death and disability worldwide (1), yet advancements in its treatment have increased medical expenditure. In 2017, China’s expenditure on cerebrovascular disease treatment reached USD$ 83.83 billion, surpassing all other diseases and accounting for 17% of the nation’s total disease treatment expenditure—equivalent to 0.66% of GDP (2). Cerebral infarction (CI) is the most prevalent cerebrovascular condition, with increasing incidence rates and treatment costs significantly exceeding those of many other diseases. This trend imposes direct financial burdens on patients and families while stressing the basic medical insurance system (3). Managing the rapid increase of CI-related healthcare costs has become a critical challenge for China’s healthcare system, which is also an urgent issue worldwide (4).

The Chinese healthcare department has proactively tackled cost containment through payment reforms. In 2018, Beijing implemented the Diagnosis-Related Groups (DRG) payment system. Building upon this pilot initiative, the China Healthcare Security Diagnosis-Related Groups (CHS-DRG) was formally established in 2019 as the national standard. This establishment marks a significant milestone in the development of China’s healthcare financing mechanisms, with immediate expansion to 30 major cities (5). This CHS-DRG provides standardized grouping protocols and reimbursement benchmarks, enabling systematic cost control while ensuring the quality of care (6). The DRG payment system is expected to alleviate the increasing trend of healthcare expenditure. In this context, CI, characterized by a persistent increase in medical costs, serves as a critical use case for refining the DRG grouping method.

The DRG payment system is one of the most cutting-edge methods in global healthcare finance, becoming increasingly favored for reimbursing hospitals amid rising medical expenditure since its inception in 1983 (7, 8). In a DRG payment system, patients who exhibit analogous clinical profiles and necessitate similar levels of care are categorized under identical case types or ‘groups’, ensuring that individuals within the same DRG group consume similar healthcare resources (9). Consequently, the medical insurance provider pays money based on the DRG group to which the patient belongs and the hospital bears any costs that exceed the payment standard of the group. This framework encourages hospitals to actively engage in cost management and control. However, CHS-DRGs are developed based on data sourced from China’s first-tier cities, with a lack of research addressing the specific needs of less developed cities in the western regions. Given China’s vast territorial expanse, the economic disparities, and the diverse disease landscape, a one-size-fits-all approach to DRG implementation is impractical for developing cities (2, 10). The challenge lies in making a DRG grouping scheme adapted to the local condition (10).

Several studies have highlighted the pivotal role of comorbidity and complications (CCs) in shaping the DRG grouping strategy (10). The method for identifying CCs for the CHS-DRG draws inspiration from the DRG of the United States and Australia. This classification process involves the following two steps:

(1) Initially, CCs are ranked in descending order according to their incidence rate. Those with an incidence rate below 5% are merged into a non-CC type.

(2) Subsequently, after their weights are adjusted, taking into account the hospitalization costs and the age of the patients, CCs are classified into three types: major (MCC), moderate (mCC), and non-CC (11).

However, the analysis of the data from the target city’s data reveals a notable contrast in the incidence rates of CCs of the CHS-DRG. Given the expansive geographical spread and the resultant variation in the disease spectrum, a unified grouping standard like CHS-DRG may not be well suited to the local region. Therefore, there is an urgent need to adapt CC classification criteria to accommodate the unique healthcare characteristics of different cities, ensuring systematic alignment with local disease patterns.

This study examines the rising trends in medical expenditures and introduces a novel two-stage DRG framework designed to mitigate these costs, focusing specifically on a developing city in western China. Our research is centered on CI, which has been observed to have the most rapid increase in medical expenditures. Based on the collected data, the proposed two-stage method is designed to better align with the local context and disease spectrum. In the first stage of the method, regression analysis is used to classify CCs that occur in the city. In the second stage, decision tree ID3, a machine learning algorithm, is used to group patients with similar medical expenses. The performance of the two-stage method is compared with the grouping method of the CHS-DRG. This study fills a gap in the extant literature by focusing on a developing city and proposing feasible rules for the DRG grouping in different regions according to local conditions.

The rest of this article is organized as follows: The “Materials and Methods” section introduces the materials and methods necessary for developing the two-stage method. The “Results” section first presents the grouping results derived from the benchmark method CHS-DRGs and then the step-by-step results of our proposed two-stage model. This section also shows a comprehensive comparison of results obtained through these two approaches. The “Results” section is followed by “Discussion” and “Conclusion” sections. The limitation of this study is discussed in the “Conclusion” section.

## 2 Materials and methods

**Figure 1:**
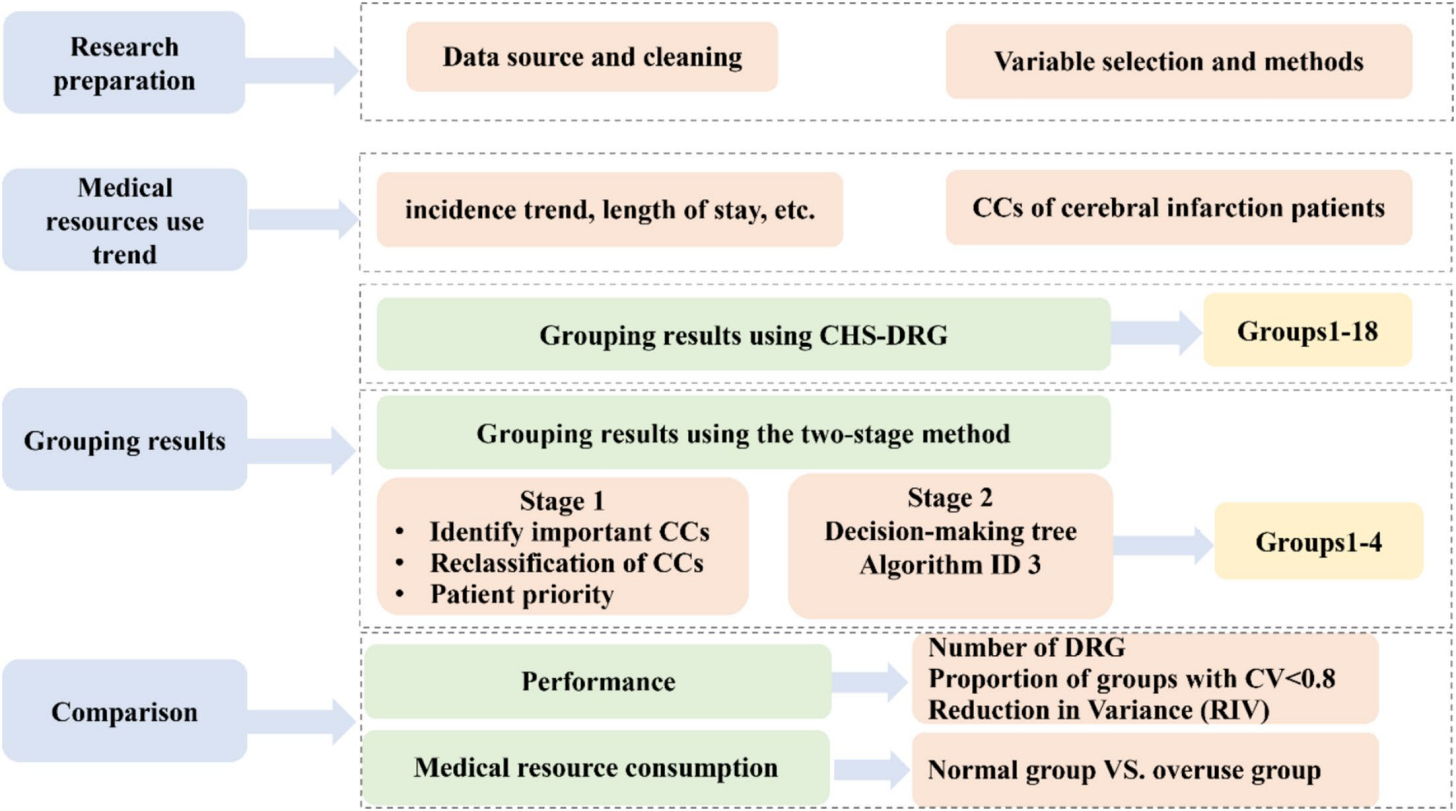
Research roadmap.

The research roadmap is shown in Figure 1.

(1) The first step was the research preparation, where we introduced the data sources, data processing, and methods applied.

(2) The second step was descriptive statistics on the data of this sample from the aspects of morbidity, hospitalization resource consumption, etc.

(3) The third step was to show our two-stage method and the grouping results. In this step, the CHS-DRG is compared as the benchmark. Thus, the following two substeps are included:

(a) First, we applied the existing CHS-DRGs classification criteria to group patient cases collected from the target area, where CCs were classified with the China Healthcare Security-Technical Specification (CTS) (12), as shown in Supplementary Figure A1.
(b) Second, we developed the novel two-stage method:

- In the first stage, we constructed seven regression models based on seven diagnoses of CCs with other control variables such as age, sex, and insurance type to identify CCs with a significant impact on medical expenses under each diagnosis.
- In the second stage, we applied a decision tree algorithm to obtain the grouping plan and improve the adaptability of the DRG, which can contribute to better cost control.

(4) In the final step, we evaluated the performance of the two-stage method by comparing it with the CHS-DRGs regarding grouping results and the utilization of medical resource consumption.

### 2.1 Patient data

The dataset for this research was rigorously collected from the Healthcare Security Administration of the target city (HSAC) in western China. The data span a period of 3 years (2016–2018), providing a longitudinal perspective on healthcare trends and patterns. The dataset encompasses a substantial sample size of 111,025 inpatient cases with CI (the International Classification of Diseases, Tenth Revision, Clinical Modification [ICD-10] codes I63.1–I63.9) as the main diagnosis from 416 hospitals. The original dataset includes 58 variables, including sex, age, length of stay (LOS) in hospital, cost of hospitalization, insurance type, and seven CCs. We claim that the data are non-public, and all experimental protocols have been approved by the HSAC.

### 2.2 Data cleaning and variable selection

Initially, 111,025 patient cases were collected, with incomplete data excluded during preprocessing. Further refinement excluded extreme cases with LOS over 60 days, aligning with a procedure referenced in Ref. [13], resulting in 109,375 cases. Considering the annual medical insurance reimbursement limit of USD$ 22,189 by the city’s policy, cases with hospitalization expenditures exceeding this limit were treated as outliers and excluded, leading to a final refined dataset of 109,314 cases.

Next, in addition to CCs, this study included six control variables resulting from our previous study (3): sex, age, LOS, hospital level, insurance type, and payment level.

### 2.3 Grouping method

CCs have a significant impact on medical expenditures (14). An effective classification of CCs is vital to establishing a scientific and reasonable DRG grouping plan (15). In the CHS-DRG, CCs with higher incidence rates are ranked higher when classifying CCs. However, CCs with high incidence rates do not always produce high medical expenses. Since the goal of implementing the DRGs is to control medical expenses, CCs should be classified according to their impact on medical expenses. For this purpose, regression analysis was adopted in the first stage of the method.

Regression analysis is a statistical method commonly used to determine whether there is a dependence between two variables, and the degree of dependence can be indicated by the coefficients (16). Therefore, the *p*-values of the variables were used to test the significance of CCs. The regression coefficient of significant CCs was used to quantify their impact on medical expenses. Specifically, we constructed seven regression models corresponding to the seven diagnoses on CCs, as suggested by the staff of HSAC that the principal diagnosis, i.e., CI, and seven primary diagnoses on CCs are valuable if the patient has multiple CCs. We analyzed the significance of CCs and obtained the regression coefficients for each model. Those CCs that were significant (*p* < 0.05) in at least one model were selected and then ranked with their regression coefficients.

Description of the models are described in Table 1, and the seven regression models, indexed by i from 1 to 7, respectively, are shown as Equation (1):


(1) $$y=\sum_{j=1}^{J_{i}}\beta_{ij}*x_{ij}+\sum_{n=1}^{N}\gamma_{n}*{\text{control}}_{n}+e,\;i=1,\ldots ,7$$

**Table 1:** Description of the regression models.

Input			Output	
Dependent variable indicating medical expense	Independent variable indicating CCs	Control variable indicating age	Regression coefficient $\beta_{ij}$	*p*-value of $x_{ij}$
y	$x_{ij}\in \left\{0,1\right\}$	${\text{control}}_{n}$		

In these models, the dependent variable y represents the medical expense of each patient. The independent variable $x_{ij}$ corresponds to the jth CC in the ith diagnosis/model, which is a 0,1-variable indicating whether a patient has this CC. The number of CCs in each diagnosis i is denoted as $J_{i}$. The regression coefficient of $x_{ij}$ is $\beta_{ij}$. These seven models also include N=6 control variables, ${\text{control}}_{n}\;\left(n=1,\ldots ,N\right)$, representing age and sex, among others, as discussed in the “Grouping Results using CHS-DRG as the benchmark” section. The coefficient associated with ${\text{control}}_{n}$ is $\gamma_{n}$ and e is the standard error.

The identified CCs were then classified into three types: Type I, Type II, and Type IIII. For each model, the top 30% of CCs were considered to be major, i.e., MCC; the CCs ranked 30–60% were considered moderate, i.e., mCC; and the last 40% were non-CC.

Based on the classification of CCs, patients with different CCs were classified into three types of priorities according to their type of CCs. As shown in Table 2, if a patient has at least one MCC among the seven primary diagnoses, then they are considered the highest priority, that is, type I; if they do not have any MCC but has at least one mCC, then they are supposed to be type II; otherwise, they belong to type III.

**Table 2:** Patient type based on CCs.

Patient priority	Classification of CCs		
	MCCs	mCC	Non-CC
Type I (severity)	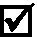	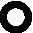	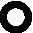
Type II (general)	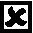	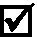	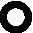
Type III (slight)	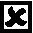	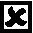	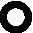

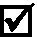
: Has this type of CCs. 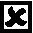: Does not have this type of CCs. 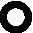: Has/has not had this type of CCs.

After selecting and ranking CCs, the decision tree classifier was applied. The decision tree classifier has been used in various clinical studies (17). An important advantage of this method is that it does not require the selection of explanatory variables prior to modeling. Furthermore, their non-parametric feature allows them to deal with missing values, and they are robust to the presence of outliers. In the second stage of the method, a decision tree, i.e., the iterative dichotomiser 3 (ID3) algorithm, was used to establish the grouping of DRG. The training set was grouped, and the effect of grouping was evaluated using the test set’s data. We conducted 10 random trials using data samples, and the results of these 10 trials were consistent, indicating the stable performance of the algorithm.

All analyses discussed above were performed using the RStudio 4.0.2, an open source software provided by Posit (18).

## 3 Results

In the “General Information about Inpatient Medical Expenditure” section, we summarize the general characteristics of the participants in the study and the results of single factor analysis. In the “Grouping Results using CHS-DRG as the benchmark” section, the grouping results of CHS-DRGs are presented. The grouping results of the proposed two-stage method are shown in the “Grouping results using the two-stage method” section. Finally, the performance of the two algorithms is presented in the “Comparison of the performance and medical expenditure overuse” section.

### 3.1 General information about inpatient medical expenditure

From 2016 to 2018, after excluding outliers, a total of USD$ 170.94 million in hospitalization expenses were incurred for patients with stroke medical insurance, with average expenses of USD$ 1564.342. Among the patients, 54,115 (49.50%) were male and 55,199 (50.50%) were female. There were 68,498 (62.66%) urban employees and 40,816 (37.34%) urban and rural residents. As the older adults (over 65 years of age) accounted for the majority of patients, the older adult population was further divided into three subgroups based on age: 65–75, 75–85, and over 85 years. There were 2,080, 27,637, 35,512, 34,175, and 9,874 patients of the ages below 65, 65–75, 75–85, and over 85 years of age in the target city, accounting for 1.9, 25.32, 32.49, 31.26, and 9.03% of the population included in the study, respectively. Among the patients, 37.97% were hospitalized for less than 9 days, 20.97% for 9–12 days, and 41.06% for more than 12 days, as shown in Table 3.

**Table 3:** General information about inpatient medical expenditure of patients with CI.

Variable definitions	Assignment of influencing factors	Simple size	Proportion (%)	Median cost (USD)
Sex	Male	54,115	49.50	1182.30
	Female employee insurance	55,199	50.50	1117.56
Type of insurance	Urban and rural resident insurance	68,498	62.66	1305.99
	Rural	40,816	37.34	880.29
Level of hospital	Primary hospital	21,463	19.63	435.80
	Secondary hospital	35,771	32.72	1032.74
	Tertiary hospital	52,080	47.64	1695.10
Level of charge	Grade A tertiary hospital	29,946	27.39	2076.27
	Grade B tertiary hospital	22,130	20.24	1269.70
	Grade A secondary hospital	28,595	26.16	1093.57
	Grade B secondary hospital	5,462	5.00	707.55
	Grade B tertiary hospital, up 10%	3,111	2.85	878.86
	Grade B tertiary hospital, down 10%	20,070	18.36	424.35
Age	Age ≤ 45 years	2,080	1.90	1318.99
	Age between 45 and 65 years	27,673	25.32	1123.59
	Age between 65 and 75 years	35,512	32.49	1126.75
	Age between 75 and 85 years	34,175	31.26	1165.11
	Age ≥ 85 years	9,874	9.03	1206.43
LOS	≤9 days	41,510	37.97	727.14
	9 ~ 12d	22,920	20.97	1125.06
	≥12 days	44,884	41.06	1777.6
CCs	MCC	18,469	16.90	1576.95
	CC	75,662	69.22	1129.17
	Non-CC	15,183	13.89	795.10

### 3.2 Grouping results using CHS-DRG as the benchmark

**Figure 2:**
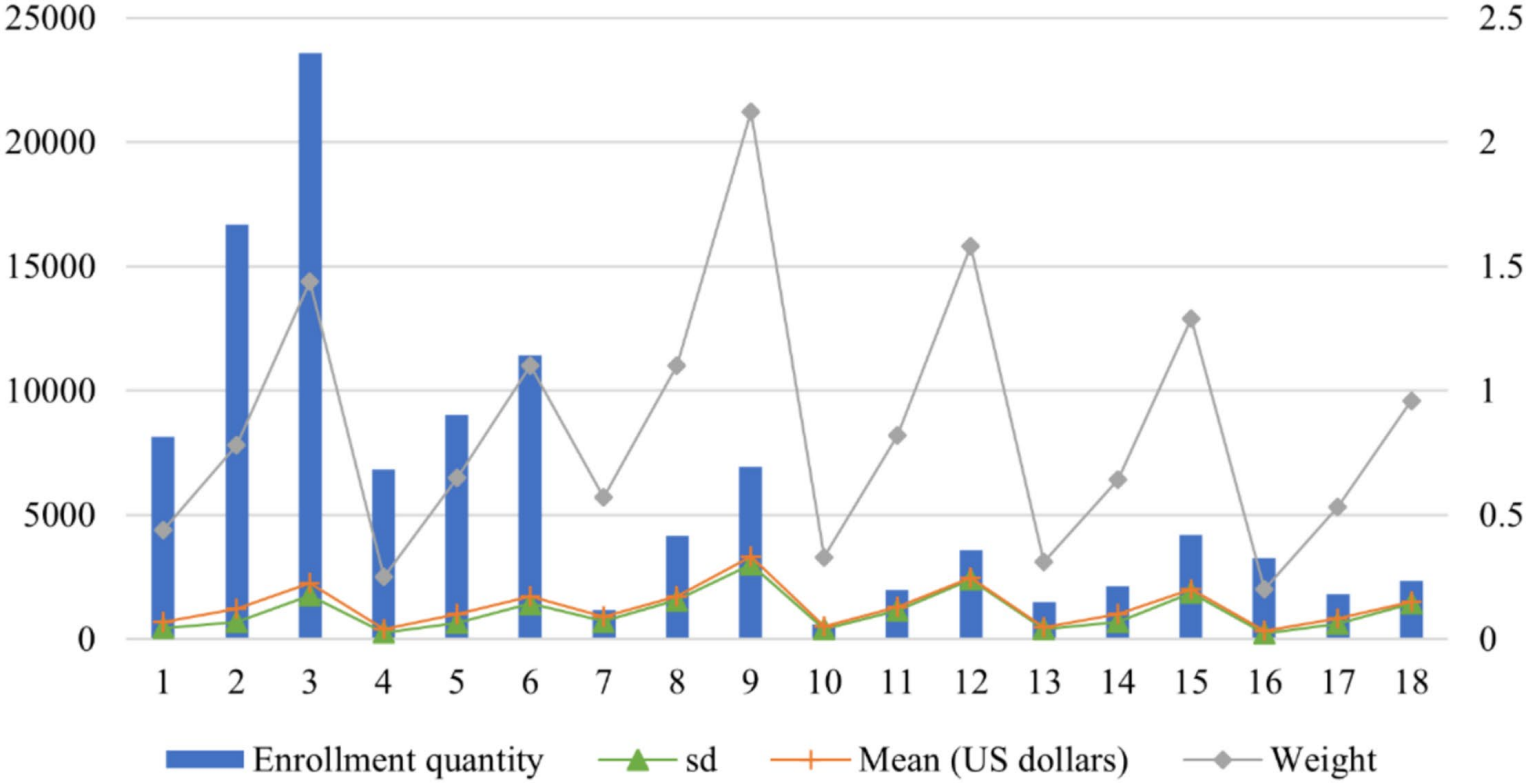
Grouping results and expenses of each group based on CHS-DRG.

To mitigate the skewness in hospitalization cost distributions, a natural logarithmic transformation was applied to normalize the data. As reported in Table 4, all the subdivided DRGs have a CV below 1, fulfilling the criteria for effective grouping. Figure 2 illustrates four key indicators: Enrollment quantity, mean cost (in USD$), standard deviation (SD), and weight. The weights ranged from a minimum of 0.20 (the 16th group) to a maximum of 2.12 (the 9th group), reflecting substantial variation in resource consumption across the disease group. Group 16, characterized by the lowest resource utilization, had an average cost of USD$ 319.18 and included 3,258 patients, accounting for 2.9% of the total cohort. Conversely, the 9th group, including patients with serious CCs in tertiary hospitals, spends the highest cost and has the highest average cost of USD$ 3,321.86.

**Table 4:** Grouping results and expenses of each group based on CHS-DRG.

Group number	Enrollment quantity	Mean	SD	CV	IQR	P75	Weight	Upper limit of expense
1	8,124	690.06	429.50	0.62	408.76	878.74	0.44	1,491.88
2	16,678	1,221.77	696.15	0.57	812.78	1,475.49	0.78	2,694.67
3	23,599	2,254.00	1,765.26	0.78	1,226.55	2,682.47	1.44	4,522.30
4	6,806	393.12	260.54	0.66	221.21	495.97	0.25	827.80
5	9,034	1,016.63	635.00	0.62	626.96	1,244.75	0.65	2,185.20
6	11,421	1,722.86	1,414.44	0.82	950.34	2,051.82	1.10	3,477.33
7	1,181	897.35	702.25	0.78	508.78	1,097.60	0.57	1,860.78
8	4,171	1,716.34	1,556.73	0.91	951.45	1,910.65	1.10	3,337.84
9	6,947	3,321.90	2,987.84	0.90	1,509.59	3,979.61	2.12	6,244.00
10	595	509.94	387.42	0.76	265.23	624.46	0.33	1,022.32
11	1,974	1,287.61	1,159.70	0.90	698.37	1,477.42	0.82	2,524.98
12	3,601	2,464.50	2,390.28	0.97	1,150.48	2,825.83	1.58	4,551.56
13	1,499	483.94	384.73	0.79	239.80	619.81	0.31	979.52
14	2,113	999.32	683.14	0.68	601.24	1,195.92	0.64	2,097.78
15	4,186	2,012.63	1,859.25	0.92	1,028.90	2,340.47	1.29	3,883.82
16	3,258	319.17	223.00	0.70	164.41	423.00	0.20	669.62
17	1,801	827.55	605.01	0.73	489.66	1,025.23	0.53	1,759.72
18	2,326	1,498.25	1,422.46	0.95	766.26	1,782.33	0.96	2,931.73

CV, coefficient of variation; IQR, interquartile range; SD, standard deviation.

### 3.3 Grouping results using the two-stage method

The grouping results presented in the previous section categorizes all patient cases into 18 distinct groups. Notably, six of these groups have a CV above 0.8, suggesting the necessity for refining the CHS-DRG framework. This finding is corroborated by the National Healthcare Security Administration of China, which has identified the deficiency in applying CHS-DRG across the country (19). Scholars widely agree that enhancing the management of CCs is crucial for developing a DRG system tailored to local characteristics (15).

To improve the CHS-DRG grouping method, a two-stage grouping method is proposed to regroup 109,314 patient cases with CI in the target city, which mainly included the following steps:

First stage: To (1) identify the important CCs affecting hospitalization costs and (2) classify significant CCs into three different levels to replace the three severe levels of CCs in the CHS-DRG.

Second stage: To (1) prioritize the patient cases according to the important CCs after classification and (2) regroup cases using the decision tree, algorithm ID3.

#### 3.3.1 First stage: classification of CCs

In the first stage, regression analysis is used to determine whether there is a dependence between the cost and CCs, and then, the regression coefficients are used to rank the degree of dependence. The results of the seven regression models are reported in Supplementary Tables A1–A7, and 641 significant CCs in total were identified and ranked. For example, as Supplementary Table A1 shows, G97 is one of the CCs in the regression model 1. The regression coefficient of G97 is 99,142.41 and the *p*-value was <0.05. Therefore, G97 is marked as a significant CC.

As indicated in Section 2, the identified 641 CCs were then classified into three types: MCC, mCC, and non-CC. Part of the classification results of CCs are listed in Table 5 and the complete results for the 641 CCs are listed in Supplementary Table A8. For example, the regression coefficient of G97 in model 1 ranks in the top 30%, and therefore, G97 is considered an MCC in our new classification of CCs.

**Table 5:** Part of classification results of CCs of the seven regression models.

Classification	ICD code for diseases
MCC code (top 30%)	G97, T06, B37, F43, A40, D46, B95, R23, M88…
mCC code (30–60%)	J95, B44, Q12, I12, B49, S73, A41, I85, J12, T82…
non-CC code (60–100%)	Z98, L88, G92, C25, D73, R57, D84, J69, J96, E16…

#### 3.3.2 Second stage: grouping results using the algorithm ID3

**Figure 3:**
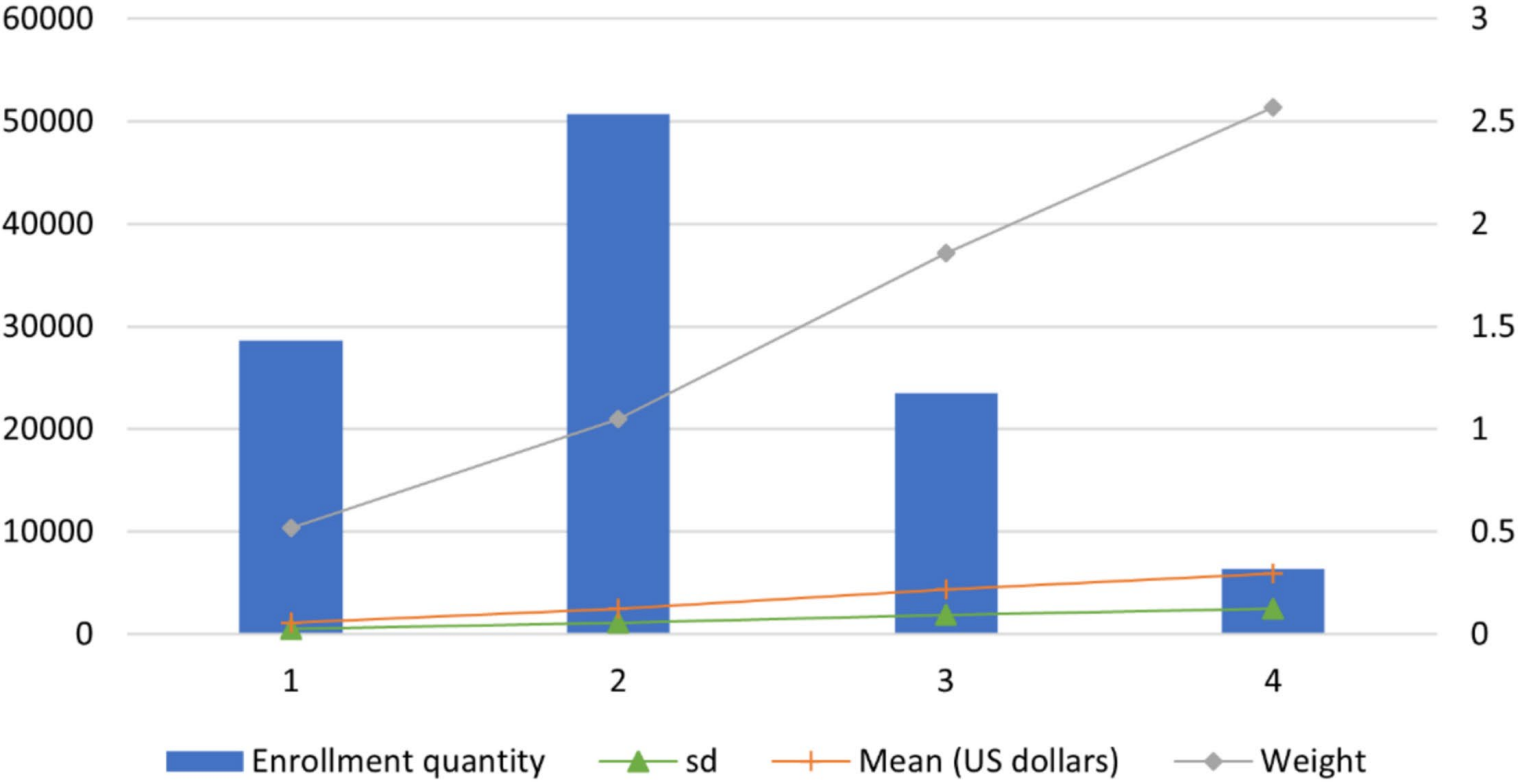
Grouping results and expenses of each group based on the two-stage method.

Employing the identified CCs and other control variables as grouping factors, along with hospitalization expense as the dependent variable, we identified the patients’ type following the rule shown in Table 2. Then, a two-stage grouping method was developed based on the algorithm ID3. After merging patient cases with less than 200, the method resulted in four distinct groups. As detailed in Table 6 and Figure 3, all groups exhibit a CV below 1, signifying a low inner group variation and adherence to DRG requirements. In the meantime, there is a notable disparity in resource consumption and medical expenses among the groups. For example, the 1st group, with the smallest weight of 0.52, comprises 28,643 patients with an average medical expense of USD$ 625.10. In contrast, the 4th group, with the highest weight of 2.57, includes 6,411 patients with an average medical expense of USD$ 3,443.37.

**Table 6:** Grouping results of the two-stage method.

Group number	Enrollment quantity	Mean	SD	CV	IQR	P75	Weight	Upper limit of expense
1	28,643	625.10	492.09	0.79	301.84	820.48	0.52	1,273.24
2	50,725	1,412.13	1,118.14	0.79	820.11	1,643.08	1.05	2,873.25
3	23,535	2,521.85	1,882.56	0.75	1,403.93	2,911.92	1.86	5,017.82
4	6,411	3,443.37	2,504.83	0.73	1,683.88	4,025.18	2.57	6,551.00

CV, coefficient of variation; IQR, interquartile range; SD, standard deviation.

### 3.4 Comparison of the performance and medical expenditure overuse

DRG grouping aims to ensure that patients with similar resource consumptions/medical expenses are put in the same group, thus reasonably controlling the medical expenses of each group. According to the evaluation guidance in the CTS, the performance of the proposed two-stage method is compared with that of the CHS-DRG, as listed in Table 7.

(1) Number of groups: The number of groups obtained by the two-stage method is four, which meets the requirements of DRG, and fewer groups (compared with CHS-DRG) lead to easier implementations.
(2) The proportion of groups whose CV is less than 0.8: CV is an important index to evaluate the grouping performance, and a smaller CV indicates smaller inter-group variation and higher intra-group homogeneity. According to CTS, a successful grouping result is obtained in groups with a CV of less than 0.8. As shown in Table 7, only 67% of groups obtained by the CHS-DRG have CVs less than 0.8. However, the CV of all groups resulting from the two-stage method is less than 0.8, which shows a significant improvement.
(3) Reduction in variance (RIV): In a decision tree, a larger RIV indicates a better grasp of the inherent law of the data and a higher degree of systematization. RIV of the CHS-DRG is 127.94%, but that of the two-stage method is 131.27%.

**Table 7:** Comparison of grouping performance between the CHS-DRG and the two-stage grouping method.

Evaluation indicators	CHS-DRG	Two stage
Number of DRG	18	4
Overall case enrollment rate	100%	100%
Standardized case enrollment rate	100%	100%
Proportion of groups whose CV is less than 1	100%	100%
Proportion of groups whose CV is less than 0.8	67%	100%
Reasonable proportion of enrolled cases	100%	100%
Reduction in variance (RIV)	127.94%	131.27%

As the implementation of DRG is aimed at controlling the medical expenses of patients in each group within a certain range (20), we demonstrate the superiority of applying the two-stage method by comparing it with the CHS-DRG regarding the overuse of medical resources, as listed in Tables 8, 9. The 75th quantile plus 1.5 times interquartile range (P75 + 1.5IQR) of each group is used to calculate the upper limit of medical expense per patient (21), listed in the last column of Tables 4, 6, respectively. A patient case that spends medical expenses less (respectively larger) than the upper limit in each group is considered to be normal (respectively overuse). All the normal (respectively overuse) cases in a group are incorporated into the normal group (respectively overuse group). The overuse rate, i.e., the proportion of patients in the overuse group, is used to measure the two grouping methods.

**Table 8:** Statistics on medical resources according to the CHS-DRG.

Group number	Number of patients				Mean (USD$)			Median of LOS		Average number of CCs	
	Total	Normal group	Overuse group	Overuse rate (%)	Normal group	Overuse group	Times	Normal group	Overuse group	Normal group	Overuse group
1	8,124	7,908	216	2.66	402.93	1,687.42	4.19	10	20	5	6
2	16,678	16,192	486	2.91	1,082.84	3,246.01	3	12	25	5	6
3	23,599	21,883	1,716	7.27	1,700.59	5,942.31	3.49	12	28	5	5
4	6,806	6,424	382	5.61	322.34	999.85	3.1	8	16	3	4
5	9,034	8,649	385	4.26	859.32	2,627.81	3.06	10	24	4	6
6	11,421	10,670	751	6.58	1,318.31	4,725.59	3.58	10	21	4	5
7	1,181	1,108	73	6.18	735.50	2,235.95	3.04	12	27	6	7
8	4,171	3,852	319	7.65	1,272.48	4,921.30	3.87	14	28	6	7
9	6,947	6,173	774	11.14	2,153.25	8,872.48	4.12	13	30	6	7
10	595	551	44	7.39	397.63	1,562.86	3.93	9	23	4	7
11	1,974	1,836	138	6.99	969.08	3,374.85	3.48	9	22	6	7
12	3,601	3,206	395	10.97	1,627.37	6,475.59	3.98	10	23	6	7
13	1,499	1,391	108	7.2	374.26	1,186.68	3.17	10	19	1	2
14	2,113	2,006	107	5.06	839.79	2,660.06	3.17	10	19	2	0
15	4,186	3,869	317	7.57	1,493.79	5,459.46	3.65	10	19	1	0
16	3,258	3,050	208	6.38	254.29	784.02	3.08	8	19	0	0
17	1,801	1,719	82	4.55	687.13	2,210.65	3.22	8	17	0	1
18	2,326	2,166	160	6.88	1,122.33	3,637.13	3.24	8	15	1	0

**Table 9:** Statistics on medical resources according to the two-stage grouping method.

Group number	Number of patients				Mean			Median of LOS		avg. Number of CCs	
	Total	Normal group	Overuse group	Overuse rate (%)	Normal group	Overuse group	Times	Normal group	Overuse group	Normal group	Overuse group
1	28,643	26,456	2,187	7.64	482.91	1,573.29	3.26	10	19	3	6
2	50,725	47,623	3,093	6.1	1,129.17	3,777.58	3.35	10	24	5	6
3	23,535	21,749	1,786	7.59	1,907.56	6,823.83	3.58	12	27	5	6
4	6,411	5,729	682	10.64	2,354.15	9,349.16	3.97	13	28	5	7

For the CHS-DRG, the highest overuse rate is observed in the 9th group, reaching 11.14% and the overuse rate of the 12th group also exceeds 10%, indicating an unbalanced grouping outcome. For the two-stage method, the overuse rates of the first three groups are all less than 8%. Though the overuse rate of the 4th group exceeds 10%, this group only includes a small proportion of patients compared to those in the fourth group. For the average medical expense, a significant difference is observed between each overuse group and the corresponding normal group by the RHS-DRG. For example, the average cost of the overuse group for the 1st and 9th groups is more than four times that of the normal group by the RHS-DRG. In contrast, the average cost of the overuse group for the 5th and 7th groups is approximately three times that of the normal group. By contrast, the difference between each overuse group and the corresponding normal group is relatively small by the two-stage method, which ranges between 3.26 and 3.97. LOS is also an important indicator to show the performance of a grouping method, as a longer time for a patient staying in the hospital usually leads to more resource assumptions. LOS can also implicate the use of resources that cannot be directly reflected by money. These two methods show similar performance on LOS.

## 4 Discussion

The increase in medical expenses in China has outpaced the average growth rate of the national GDP. In the target city, focusing on ischemic stroke (CI), the patient population increased sharply from 22,672 to 55,081 between 2011 and 2018. Concurrently, the average expenditure per patient escalated from USD$ 928 in 2011 to USD$ 1,081 in 2018. This significant increase in medical expenses has imposed a substantial financial burden on the healthcare insurance system. Controlling medical costs has emerged as a critical priority for policymakers. The DRG system, recognized as a sophisticated approach to medical payment management, aims to mitigate inefficiencies and reduce expenditures. This method was initially implemented in Beijing, the capital of China, in 2018. The Chinese government then developed the CHS-DRG and introduced it to 30 other cities. However, the unbalanced economic and regional variations in the disease spectrum pose significant challenges to the applicability of this method since the DRG system is primarily developed using data from the developed regions.

The city selected for our study is located in an economically underdeveloped region in western China. In this city, the medical expenses of CI have witnessed a sharp increase, prompting its selection as the focal point of our research. Using local healthcare data, we categorized patients into groups based on hospitalization costs and clinical profiles. To this end, a novel two-stage method devised specifically for this investigation was developed and compared to the existing CHS-DRG method, which enjoys widespread adoption in China. Our findings indicate that tailoring the classification of CCs according to local characteristics prior to grouping significantly enhances the adaptability of the DRG system. Specifically, in the target city, implementing the two-stage method for DRG could result in savings of approximately USD$ 8.71 million. If implemented nationally, this method can potentially alleviate the financial burden on China’s healthcare system.

CHS-DRG, recognized as China’s most authoritative DRG grouping system (4), has been developed by applying the Australian case-mix classification method with data from developed regions in China (22). The severity of CCs is determined by their incidence rates. CCs with high incidence rate are considered severe, while those with low incidence are considered general. Our research in a western Chinese city revealed that the CHS-DRG system, while comprehensive, resulted in complex and numerous groups of cases. The incidence rate of CCs in the city is significantly different from the classification in the CHS-DRG, which may be due to the significant regional variance in the disease spectrum since the vast expanse of China leads to the diversity in regional dietary habits and economic disparities. Consequently, the CHS-DRG system may not uniformly apply across the country. It is imperative that each region tailors its DRG grouping strategy to reflect local epidemiological patterns and healthcare needs.

Empirical evidence consistently demonstrates that CCs are significant determinants of healthcare expenditures (23). The findings of this study highlight that the incidence rates of CCs exhibit substantial regional disparities. Consequently, applying a uniform set of criteria across the entire country would be imprudent. The fundamental aim of DRG implementation is to optimize cost management in the healthcare system. Therefore, when developing DRG grouping criteria, it is imperative to prioritize the impact of CCs on medical expenditures over their incidence rates. Utilizing data from the local region, we constructed seven regression models in the first stage to classify CCs and, subsequently, in the second stage, grouped the patient cases based on algorithm ID3. This two-stage method significantly enhanced the performance of CCs classification.

The grouping result of the two-stage method is reported in Table 6 and that of the CHS-DRG is presented in Table 4. The CHS-DRG obtains 18 groups, but only 4 groups resulted from the two-stage method, which indicates that the two-stage method is notably straightforward, making it easily implementable across a wide range of regions, thereby underscoring its robust practical applicability. These results also suggest that different classifications of CCs can lead to different grouping plans. According to Table 7, the two-stage method significantly improved the CHS-DRG in terms of the proportion of groups with CV less than 80%, from 67 to 100%. The smaller intra group difference of the two-stage method indicates it to be a more reasonable grouping plan for the DRG in the target city.

We used the P75 + 1.5IQR as the upper limit of each group, listed in Tables 4, 6 to identify outliers within each group, categorizing these outliers as instances of medical overuse. Based on the two proposed models, the medical resource utilizations between the normal and overuse groups are shown in Tables 8, 9, respectively. The medical resources under consideration encompassed several key metrics: the number of patients, average medical expense, and the LOS. Notably, the LOS for the overuse group is significantly more significant compared to the normal group, indicating a potential avenue for cost reduction through LOS management.

Tables 8, 9 also present the average number of CCs in each group for the two methods. This reveals a substantial disparity between the normal and overuse groups within the same DRG group, underscoring the variability in medical resource allocation. In the context of the two-stage method, more CCs are observed in overuse groups than in the corresponding normal group, which aligns with the medical expenses of each group, verifying the importance of considering CCs in assessing medical resource utilization (24). An anomaly is observed in the CHS-DRG grouping results, specifically within the 14th, 15th, and 18th groups. Contrary to expectations, the overuse group exhibited fewer CCs than the corresponding normal group, detailed in Table 8, which may be because the CHS-DRG classifies CCs based on the incidence rate and thus some CCs having low incidence rates but high costs are ignored. However, medical expenses, not the incidence rate, matter when developing DRG. For example, hypertension is a common CC of CI, but the cost of treating hypertension is very low, which also implicates the need for improvement in the CHS-DRG.

Furthermore, the average medical expense and number of patients for each overuse group reported in Tables 8, 9 and the upper limit expenditure for each group in Tables 1, 5 can contribute to the following insights: Should a stringent full control policy be enforced to curb medical expenditures, it is projected that the implementation of the CHS-DRG would result in an estimated savings of USD$ 8.14 million for the target city. Furthermore, the application of our proposed two-stage method could yield even larger savings, with a projected reduction of USD$ 8.59 million in medical expenses. This outcome is desirable to the government and the medical insurance department.

## 5 Conclusion

Although the CHS-DRG system is recognized as China’s most authoritative medical payment, our study indicates that regional customization is essential. CCs should be tailored according to data from local regions rather than adhering to a one-size-fits-all DRG system nationwide. In this research, we used real data from a developing city to identify suitable methods for DRG grouping, thus filling a gap in the extant literature only considers developed regions. We developed a two-stage grouping method through the classification of CCs. The effectiveness of the method was demonstrated using real data, and its performance was analyzed via the comparison with the CHS-DRG.

The scope of this study is limited by the use of data from a single city. To enhance the generalizability of our findings, it is recommended that data from a minimum of three cities be included in future research. Additionally, the absence of a standardized surgical coding system across hospitals has restricted our ability to incorporate surgical codes into the analysis. This finding highlights the necessity for further research that addresses these methodological constraints and expands the scope of our understanding.

## Data Availability

The datasets presented in this article are not readily available because the data was taken from the Medical Insurance Laboratory of the Healthcare Security Administration of Chengdu and there are administration restrictions. Requests to access the datasets should be directed to the corresponding author.
