# Supplementary material for: Two-stage DRG grouping of cerebral infarction based on comorbidity and complications classification
Source: Front Public Health. 2025 Apr 28;13:1513744. doi: 10.3389/fpubh.2025.1513744 (PMC12066792; doi:10.3389/fpubh.2025.1513744)
Supplement: Supplementary file 1 [file Table_1.DOCX]

Supplementary Material

# Supplementary Figures and Tables

## Supplementary Figures


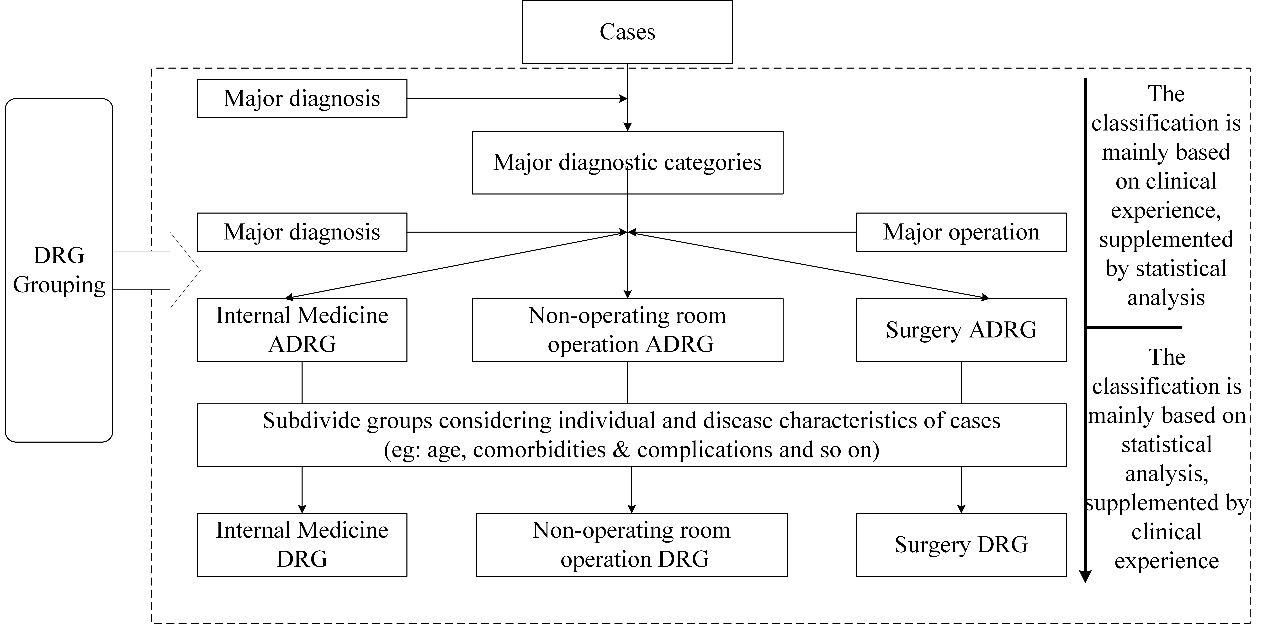


**Figure A1.** The CHS-DRG grouping

## Supplementary Tables

**Table A1.** Regression analysis results of Model 1

(only present CCs with P-value < 0.05)

| ICD code of first CCs | Estimate | Std.. Error | t. value | P-value |
| --- | --- | --- | --- | --- |
| A40 | 53511.84 | 16079.71 | 3.33 | 9.00E-04 |
| A41 | 40648.33 | 11492.99 | 3.54 | 4.00E-04 |
| B37 | 67038.76 | 12281.08 | 5.46 | 0 |
| B44 | 43524.24 | 12455.29 | 3.49 | 5.00E-04 |
| B49 | 41909.53 | 11742.96 | 3.57 | 4.00E-04 |
| B95 | 49073.37 | 16079.71 | 3.05 | 0.0023 |
| C25 | 29131.28 | 12281.08 | 2.37 | 0.0177 |
| D46 | 49526.91 | 13925.43 | 3.56 | 4.00E-04 |
| D73 | 28685.79 | 13925.43 | 2.06 | 0.0394 |
| D84 | 26496.84 | 12455.29 | 2.13 | 0.0334 |
| F43 | 63325.25 | 13129.02 | 4.82 | 0 |
| G92 | 29760.21 | 13925.43 | 2.14 | 0.0326 |
| G97 | 99142.41 | 16079.71 | 6.17 | 0 |
| I12 | 43097.79 | 12712.12 | 3.39 | 7.00E-04 |
| I85 | 39384.77 | 16079.71 | 2.45 | 0.0143 |
| J12 | 37543.14 | 16079.71 | 2.33 | 0.0196 |
| J69 | 25666.36 | 11440.91 | 2.24 | 0.0249 |
| J95 | 43748.59 | 16079.71 | 2.72 | 0.0065 |
| J96 | 25359.38 | 11416.20 | 2.22 | 0.0263 |
| L88 | 32230.19 | 16079.71 | 2.00 | 0.045 |
| M88 | 45885.66 | 13925.43 | 3.30 | 0.001 |
| Q12 | 43363.90 | 16079.71 | 2.70 | 0.007 |
| R23 | 47681.41 | 13925.43 | 3.42 | 6.00E-04 |
| R57 | 28611.41 | 11457.20 | 2.50 | 0.0125 |
| R65 | 44927.21 | 12712.12 | 3.53 | 4.00E-04 |
| S73 | 41067.22 | 13925.43 | 2.95 | 0.0032 |
| T06 | 95674.61 | 16079.71 | 5.95 | 0 |
| T82 | 34712.28 | 11985.10 | 2.90 | 0.0038 |
| Z43 | 34422.83 | 13925.43 | 2.47 | 0.0134 |
| Z98 | 33255.02 | 12155.11 | 2.74 | 0.0062 |

**Table A2.** Regression analysis results of Model 2

(only present CCs with P-value < 0.05)

| ICD code of second CCs | Estimate | Std.. Error | t. value | P value |
| --- | --- | --- | --- | --- |
| A04 | 15696.22 | 2093.82 | 7.50 | 0 |
| C16 | 6728.06 | 2780.16 | 2.42 | 0.0155 |
| C91 | 20478.06 | 8101.07 | 2.53 | 0.0115 |
| D61 | 6866.86 | 2959.49 | 2.32 | 0.0203 |
| D64 | 3488.01 | 547.65 | 6.37 | 0 |
| D76 | 44687.04 | 11456.22 | 3.90 | 1.00E-04 |
| D84 | 14784.88 | 5124.13 | 2.89 | 0.0039 |
| E10 | 6907.78 | 1276.62 | 5.41 | 0 |
| E14 | 3554.00 | 227.48 | 15.62 | 0 |
| E23 | 8658.25 | 4051.42 | 2.14 | 0.0326 |
| E77 | 7463.48 | 822.04 | 9.08 | 0 |
| E78 | 1417.68 | 220.26 | 6.44 | 0 |
| E86 | 10541.81 | 5124.13 | 2.06 | 0.0397 |
| F00 | 16413.32 | 4330.99 | 3.79 | 2.00E-04 |
| F01 | 4268.38 | 747.45 | 5.71 | 0 |
| F06 | 6908.14 | 1204.87 | 5.73 | 0 |
| G08 | 17804.02 | 8101.07 | 2.20 | 0.028 |
| G20 | 1829.04 | 631.66 | 2.90 | 0.0038 |
| H35 | 6650.96 | 2701.93 | 2.46 | 0.0138 |
| I08 | 4728.34 | 1791.77 | 2.64 | 0.0083 |
| I09 | 10049.02 | 817.95 | 12.29 | 0 |
| I10 | 2464.08 | 131.70 | 18.71 | 0 |
| I11 | 1580.11 | 231.87 | 6.81 | 0 |
| I15 | 3895.12 | 1402.97 | 2.78 | 0.0055 |
| I25 | 1693.65 | 187.00 | 9.06 | 0 |
| I27 | 1345.46 | 493.89 | 2.72 | 0.0064 |
| I31 | 5430.33 | 2129.54 | 2.55 | 0.0108 |
| I33 | 52935.31 | 5728.74 | 9.24 | 0 |
| I35 | 5081.89 | 2340.45 | 2.17 | 0.0299 |
| I38 | 3015.78 | 758.45 | 3.98 | 1.00E-04 |
| I42 | 5794.94 | 1317.71 | 4.40 | 0 |
| I44 | 5087.89 | 1185.62 | 4.29 | 0 |
| I46 | 33737.42 | 4051.42 | 8.33 | 0 |
| I66 | 8328.87 | 429.60 | 19.39 | 0 |
| I70 | 3423.75 | 230.16 | 14.88 | 0 |
| I71 | 4877.66 | 2444.35 | 2.00 | 0.046 |
| I72 | 6132.15 | 1106.67 | 5.54 | 0 |
| J06 | -2051.98 | 686.78 | -2.99 | 0.0028 |
| J15 | 12341.91 | 690.37 | 17.88 | 0 |
| J18 | 12890.32 | 399.02 | 32.31 | 0 |
| J69 | 17306.83 | 1402.97 | 12.34 | 0 |
| J94 | 10497.35 | 1072.73 | 9.79 | 0 |
| J96 | 21474.63 | 661.10 | 32.48 | 0 |
| J98 | 7486.58 | 231.83 | 32.29 | 0 |
| K27 | 5042.87 | 1363.07 | 3.70 | 2.00E-04 |
| K35 | 14874.13 | 5124.13 | 2.90 | 0.0037 |
| K36 | 14935.62 | 6614.74 | 2.26 | 0.024 |
| K56 | 4886.83 | 1996.60 | 2.45 | 0.0144 |
| K65 | 6261.19 | 2865.62 | 2.18 | 0.0289 |
| K72 | 5155.40 | 1025.24 | 5.03 | 0 |
| K75 | 28452.64 | 5728.74 | 4.97 | 0 |
| K92 | 11645.23 | 675.24 | 17.25 | 0 |
| M84 | 23195.03 | 5728.74 | 4.05 | 1.00E-04 |
| N19 | 3573.67 | 789.19 | 4.53 | 0 |
| N34 | 4107.69 | 1138.51 | 3.61 | 3.00E-04 |
| R02 | 20385.41 | 6614.74 | 3.08 | 0.0021 |
| R13 | 8607.09 | 2629.96 | 3.27 | 0.0011 |
| R57 | 21317.73 | 1167.27 | 18.26 | 0 |
| R65 | 17843.17 | 4330.99 | 4.12 | 0 |
| T82 | 25348.57 | 4330.99 | 5.85 | 0 |
| Z49 | 55764.29 | 11456.22 | 4.87 | 0 |

**Table A3.** Regression analysis results of Model 3

(only present CCs with P-value < 0.05)

| ICD code of third CCs | Estimate | Std.. Error | t. value | P-value |
| --- | --- | --- | --- | --- |
| B35 | 7593.72 | 2985.43 | 2.54 | 0.011 |
| C16 | 19513.32 | 5780.85 | 3.38 | 7.00E-04 |
| C57 | 28745.31 | 11561.48 | 2.49 | 0.0129 |
| D64 | 7773.56 | 518.18 | 15 | 0 |
| E03 | 3221.12 | 1145.5 | 2.81 | 0.0049 |
| E14 | 4230.52 | 747.43 | 5.66 | 0 |
| E43 | 18928.75 | 1293.27 | 14.64 | 0 |
| E44 | 10879.5 | 3337.75 | 3.26 | 0.0011 |
| E46 | 14249.36 | 1353.79 | 10.53 | 0 |
| E53 | 9993.67 | 2185.29 | 4.57 | 0 |
| E72 | 5156.34 | 1056.22 | 4.88 | 0 |
| E79 | 2317.92 | 503.42 | 4.6 | 0 |
| F03 | 7323.7 | 1293.27 | 5.66 | 0 |
| F05 | 17555.99 | 5780.85 | 3.04 | 0.0024 |
| F20 | 5722.08 | 2652.69 | 2.16 | 0.031 |
| F41 | 3922.06 | 909.29 | 4.31 | 0 |
| F43 | 34237.3 | 11561.48 | 2.96 | 0.0031 |
| F45 | 7597.77 | 3486.14 | 2.18 | 0.0293 |
| G20 | 3757.78 | 1392.44 | 2.7 | 0.007 |
| G25 | 11822.74 | 3486.14 | 3.39 | 7.00E-04 |
| G31 | 2019.8 | 573.87 | 3.52 | 4.00E-04 |
| G41 | 77054.88 | 8175.25 | 9.43 | 0 |
| G47 | 1680.17 | 579.55 | 2.9 | 0.0037 |
| I35 | 4998.14 | 2360.32 | 2.12 | 0.0342 |
| I71 | 6047.26 | 2411.07 | 2.51 | 0.0121 |
| I83 | 3853.13 | 1806.06 | 2.13 | 0.0329 |
| J06 | 1674.99 | 814.51 | 2.06 | 0.0397 |
| J15 | 14460.48 | 2225.38 | 6.5 | 0 |
| J42 | 3141.49 | 857.99 | 3.66 | 3.00E-04 |
| J43 | 3184.64 | 912.1 | 3.49 | 5.00E-04 |
| J44 | 2938.03 | 581.72 | 5.05 | 0 |
| J63 | 59764.45 | 11561.48 | 5.17 | 0 |
| J69 | 16222.54 | 3854.02 | 4.21 | 0 |
| J84 | 6626.14 | 2411.07 | 2.75 | 0.006 |
| J94 | 10475.16 | 971.09 | 10.79 | 0 |
| J96 | 13786.16 | 1806.06 | 7.63 | 0 |
| J98 | 9057.83 | 604.92 | 14.97 | 0 |
| K06 | 34319.59 | 6675.11 | 5.14 | 0 |
| K11 | 61642.49 | 11561.48 | 5.33 | 0 |
| K27 | 12767.83 | 1875.96 | 6.81 | 0 |
| K56 | 9730.79 | 2725.36 | 3.57 | 4.00E-04 |
| K82 | 3039.9 | 1108.15 | 2.74 | 0.0061 |
| L02 | 26317.44 | 11561.48 | 2.28 | 0.0228 |
| L08 | 5926.26 | 2890.65 | 2.05 | 0.0404 |
| L29 | 6091.55 | 2890.65 | 2.11 | 0.0351 |
| L30 | 7748.21 | 1505.73 | 5.15 | 0 |
| L89 | 9386.58 | 1193.18 | 7.87 | 0 |
| M23 | 21519.9 | 4087.78 | 5.26 | 0 |
| M35 | 12050.89 | 4370 | 2.76 | 0.0058 |
| M50 | 2181.13 | 1003.35 | 2.17 | 0.0297 |
| M81 | 3982.72 | 752.13 | 5.3 | 0 |
| N20 | 1555.4 | 625.63 | 2.49 | 0.0129 |
| N39 | 10485.59 | 857.99 | 12.22 | 0 |
| R41 | 23898.43 | 5170.58 | 4.62 | 0 |
| T30 | 18429.1 | 4720.1 | 3.9 | 1.00E-04 |
| Z86 | 4627.6 | 2312.65 | 2 | 0.0454 |
| Z95 | 6668.86 | 2185.29 | 3.05 | 0.0023 |

**Table A4.** Regression analysis results of Model 4

(only present CCs with P-value < 0.05)

| ICD code of fourth CCs | Estimate | Std.. Error | t. value | P-value |
| --- | --- | --- | --- | --- |
| A18 | 32186.58 | 11539.08 | 2.79 | 0.0053 |
| A81 | 22736.33 | 11539.08 | 1.97 | 0.0488 |
| B37 | 29210.37 | 3331.52 | 8.77 | 0 |
| B44 | 52734.22 | 5769.77 | 9.14 | 0 |
| D69 | 2403.32 | 751.86 | 3.20 | 0.0014 |
| D86 | 3915.95 | 1042.11 | 3.76 | 2.00E-04 |
| E03 | 2458.77 | 879.28 | 2.80 | 0.0052 |
| E11 | 5007.49 | 226.88 | 22.07 | 0 |
| E13 | 3532.45 | 1529.51 | 2.31 | 0.0209 |
| E16 | 4674.20 | 2221.45 | 2.10 | 0.0354 |
| E79 | 2084.58 | 435.56 | 4.79 | 0 |
| E87 | 4100.70 | 250.92 | 16.34 | 0 |
| F06 | 6406.51 | 1478.59 | 4.33 | 0 |
| F07 | 10341.66 | 5160.70 | 2.00 | 0.0451 |
| G20 | 2876.95 | 822.16 | 3.50 | 5.00E-04 |
| I11 | 3299.29 | 338.85 | 9.74 | 0 |
| I21 | 8949.91 | 2647.87 | 3.38 | 7.00E-04 |
| I25 | 2729.96 | 245.80 | 11.11 | 0 |
| I26 | 21799.71 | 5160.70 | 4.22 | 0 |
| I31 | 3024.50 | 1307.86 | 2.31 | 0.0207 |
| I38 | 1700.00 | 709.96 | 2.39 | 0.0166 |
| I40 | 89262.18 | 11539.08 | 7.74 | 0 |
| I42 | 4387.01 | 1951.33 | 2.25 | 0.0246 |
| I44 | 1919.41 | 973.55 | 1.97 | 0.0487 |
| I46 | 23364.33 | 5160.70 | 4.53 | 0 |
| I48 | 9265.39 | 389.35 | 23.80 | 0 |
| I49 | 4627.13 | 317.30 | 14.58 | 0 |
| I50 | 3319.14 | 320.59 | 10.35 | 0 |
| I51 | 1899.99 | 577.82 | 3.29 | 0.001 |
| I61 | 14243.92 | 1872.79 | 7.61 | 0 |
| I66 | 6299.23 | 575.01 | 10.96 | 0 |
| I74 | 9091.21 | 1490.84 | 6.10 | 0 |
| I80 | 16247.62 | 1421.57 | 11.43 | 0 |
| I82 | 12276.42 | 2720.39 | 4.51 | 0 |
| I83 | 3528.24 | 1702.35 | 2.07 | 0.0382 |
| J18 | 10006.25 | 545.36 | 18.35 | 0 |
| J42 | 1586.14 | 469.41 | 3.38 | 7.00E-04 |
| J43 | 1492.29 | 598.00 | 2.50 | 0.0126 |
| J94 | 9216.23 | 905.73 | 10.18 | 0 |
| J98 | 7617.85 | 300.76 | 25.33 | 0 |
| K27 | 10546.41 | 1601.25 | 6.59 | 0 |
| K28 | 30451.32 | 11539.08 | 2.64 | 0.0083 |
| K31 | 2290.00 | 963.39 | 2.38 | 0.0175 |
| K35 | 25753.94 | 11539.08 | 2.23 | 0.0256 |
| K59 | 3354.82 | 556.40 | 6.03 | 0 |
| K60 | 32641.95 | 8159.47 | 4.00 | 1.00E-04 |
| K76 | 2573.68 | 259.34 | 9.92 | 0 |
| K80 | 1943.92 | 371.28 | 5.24 | 0 |
| L27 | 31557.14 | 11539.08 | 2.73 | 0.0062 |
| L50 | 4964.52 | 2308.54 | 2.15 | 0.0315 |
| M80 | 4146.42 | 828.43 | 5.01 | 0 |
| M81 | 2210.41 | 566.80 | 3.90 | 1.00E-04 |
| N25 | 8105.09 | 2221.45 | 3.65 | 3.00E-04 |
| N30 | 7156.09 | 2009.54 | 3.56 | 4.00E-04 |
| N40 | 2121.19 | 299.26 | 7.09 | 0 |
| R13 | 20192.91 | 3479.62 | 5.80 | 0 |
| R47 | 9038.72 | 4080.05 | 2.22 | 0.0267 |
| R63 | 15827.48 | 4361.70 | 3.63 | 3.00E-04 |
| T85 | 28337.90 | 11539.08 | 2.46 | 0.0141 |
| Z98 | 15968.11 | 4080.05 | 3.91 | 1.00E-04 |

**Table A5.** Regression analysis results of Model 5

(only present CCs with P-value < 0.05)

| ICD code of fifth CCs | Estimate | Std.. Error | t. value | P-value |
| --- | --- | --- | --- | --- |
| A04 | 21324.27 | 1361.46 | 15.66 | 0 |
| A41 | 32904.05 | 2981.20 | 11.04 | 0 |
| A49 | 2946.60 | 1316.58 | 2.24 | 0.0252 |
| B02 | 4220.13 | 1491.24 | 2.83 | 0.0047 |
| B16 | 5215.91 | 2407.72 | 2.17 | 0.0303 |
| B35 | 4534.54 | 2309.45 | 1.96 | 0.0496 |
| B37 | 31268.83 | 4081.90 | 7.66 | 0 |
| B44 | 56689.53 | 6665.40 | 8.51 | 0 |
| B49 | 32420.04 | 2981.20 | 10.87 | 0 |
| C22 | 11686.05 | 4363.69 | 2.68 | 0.0074 |
| C34 | 7402.60 | 2108.33 | 3.51 | 4.00E-04 |
| D07 | 27612.50 | 6665.40 | 4.14 | 0 |
| D18 | 4323.51 | 1601.72 | 2.70 | 0.0069 |
| D37 | 6909.94 | 2886.56 | 2.39 | 0.0167 |
| D38 | 10995.21 | 3481.16 | 3.16 | 0.0016 |
| D41 | 26859.57 | 11544.59 | 2.33 | 0.02 |
| D44 | 6516.31 | 3202.26 | 2.03 | 0.0419 |
| D50 | 7513.78 | 1467.02 | 5.12 | 0 |
| D56 | 11131.26 | 3202.26 | 3.48 | 5.00E-04 |
| D64 | 4674.51 | 455.26 | 10.27 | 0 |
| D68 | 13676.45 | 2264.62 | 6.04 | 0 |
| D69 | 2849.26 | 803.98 | 3.54 | 4.00E-04 |
| D86 | 3918.65 | 987.60 | 3.97 | 1.00E-04 |
| E01 | 25839.38 | 8163.34 | 3.17 | 0.0015 |
| E03 | 4863.55 | 864.34 | 5.63 | 0 |
| E04 | 2513.60 | 575.86 | 4.37 | 0 |
| E05 | 3222.03 | 1601.72 | 2.01 | 0.0443 |
| E06 | 5782.59 | 2519.72 | 2.29 | 0.0217 |
| E07 | 5980.35 | 2886.56 | 2.07 | 0.0383 |
| E10 | 9025.57 | 2222.31 | 4.06 | 0 |
| E11 | 6094.20 | 296.23 | 20.57 | 0 |
| E14 | 4057.50 | 411.76 | 9.85 | 0 |
| E23 | 18978.62 | 3332.99 | 5.69 | 0 |
| E27 | 6533.72 | 2264.62 | 2.89 | 0.0039 |
| E41 | 18076.60 | 1101.88 | 16.41 | 0 |
| E43 | 13083.79 | 963.36 | 13.58 | 0 |
| E44 | 20014.21 | 3481.16 | 5.75 | 0 |
| E46 | 14376.26 | 994.87 | 14.45 | 0 |
| E53 | 7491.07 | 1849.28 | 4.05 | 1.00E-04 |
| E55 | 8630.19 | 2886.56 | 2.99 | 0.0028 |
| E72 | 4040.59 | 871.65 | 4.64 | 0 |
| E77 | 11804.17 | 457.00 | 25.83 | 0 |
| E78 | 2240.09 | 220.18 | 10.17 | 0 |
| E79 | 1857.16 | 427.36 | 4.35 | 0 |
| E86 | 39683.75 | 8163.34 | 4.86 | 0 |
| E87 | 4927.63 | 244.16 | 20.18 | 0 |
| F01 | 6237.40 | 1161.36 | 5.37 | 0 |
| F03 | 4068.94 | 1055.07 | 3.86 | 1.00E-04 |
| F06 | 6807.29 | 1873.44 | 3.63 | 3.00E-04 |
| F09 | 10357.11 | 3481.16 | 2.98 | 0.0029 |
| F20 | 4523.20 | 1924.74 | 2.35 | 0.0188 |
| F31 | 24357.67 | 8163.34 | 2.98 | 0.0028 |
| F32 | 6608.83 | 894.76 | 7.39 | 0 |
| F41 | 4042.03 | 726.11 | 5.57 | 0 |
| G12 | 47290.33 | 11544.59 | 4.10 | 0 |
| G20 | 3122.66 | 902.88 | 3.46 | 5.00E-04 |
| G30 | 4460.11 | 1478.98 | 3.02 | 0.0026 |
| G37 | 5064.49 | 1898.57 | 2.67 | 0.0076 |
| G40 | 11438.26 | 1077.71 | 10.61 | 0 |
| G41 | 22486.05 | 6665.40 | 3.37 | 7.00E-04 |
| G47 | 1093.19 | 486.58 | 2.25 | 0.0247 |
| G62 | 5375.04 | 1455.34 | 3.69 | 2.00E-04 |
| G72 | 26779.76 | 8163.34 | 3.28 | 0.001 |
| G90 | 13743.78 | 4363.69 | 3.15 | 0.0016 |
| G91 | 7568.58 | 1803.65 | 4.20 | 0 |
| G93 | 5266.43 | 1253.19 | 4.20 | 0 |
| G96 | 124887.16 | 11544.59 | 10.82 | 0 |
| H02 | 8625.45 | 3848.49 | 2.24 | 0.025 |
| H11 | 5391.45 | 2074.06 | 2.60 | 0.0093 |
| H26 | 2465.09 | 1025.64 | 2.40 | 0.0162 |
| I05 | 11216.54 | 2581.92 | 4.34 | 0 |
| I09 | 18268.19 | 2010.27 | 9.09 | 0 |
| I10 | 5008.96 | 233.23 | 21.48 | 0 |
| I11 | 4122.85 | 446.22 | 9.24 | 0 |
| I13 | 30134.59 | 8163.34 | 3.69 | 2.00E-04 |
| I20 | 2823.89 | 1308.13 | 2.16 | 0.0309 |
| I21 | 8386.41 | 3202.26 | 2.62 | 0.0088 |
| I24 | 19051.93 | 2981.20 | 6.39 | 0 |
| I25 | 3327.93 | 321.13 | 10.36 | 0 |
| I26 | 13630.84 | 4713.28 | 2.89 | 0.0038 |
| I27 | 3890.94 | 715.00 | 5.44 | 0 |
| I38 | 1844.83 | 803.98 | 2.29 | 0.0218 |
| I42 | 9262.52 | 2721.53 | 3.40 | 7.00E-04 |
| I47 | 4671.77 | 1702.89 | 2.74 | 0.0061 |
| I48 | 10063.02 | 526.09 | 19.13 | 0 |
| I49 | 4579.90 | 376.18 | 12.17 | 0 |
| I50 | 3419.14 | 371.64 | 9.20 | 0 |
| I60 | 30830.71 | 4713.28 | 6.54 | 0 |
| I61 | 14114.85 | 2264.62 | 6.23 | 0 |
| I65 | 2866.23 | 292.36 | 9.80 | 0 |
| I66 | 6333.42 | 688.09 | 9.20 | 0 |
| I67 | 2601.32 | 296.60 | 8.77 | 0 |
| I69 | 4331.66 | 651.38 | 6.65 | 0 |
| I70 | 2781.43 | 209.99 | 13.25 | 0 |
| I71 | 9755.42 | 2800.40 | 3.48 | 5.00E-04 |
| I72 | 4911.38 | 1421.92 | 3.45 | 6.00E-04 |
| I74 | 6853.37 | 1633.41 | 4.20 | 0 |
| I77 | 6834.92 | 987.60 | 6.92 | 0 |
| I80 | 10960.29 | 1211.24 | 9.05 | 0 |
| I82 | 16674.39 | 2357.05 | 7.07 | 0 |
| I84 | 5153.60 | 1543.52 | 3.34 | 8.00E-04 |
| I95 | 11247.68 | 3848.49 | 2.92 | 0.0035 |
| J04 | 46332.69 | 11544.59 | 4.01 | 1.00E-04 |
| J13 | 27891.51 | 11544.59 | 2.42 | 0.0157 |
| J15 | 15199.13 | 1443.94 | 10.53 | 0 |
| J18 | 8482.59 | 720.49 | 11.77 | 0 |
| J37 | 7912.06 | 2886.56 | 2.74 | 0.0061 |
| J38 | 18630.02 | 8163.34 | 2.28 | 0.0225 |
| J39 | 8215.24 | 3848.49 | 2.13 | 0.0328 |
| J42 | 2277.76 | 531.53 | 4.29 | 0 |
| J44 | 2773.96 | 365.67 | 7.59 | 0 |
| J45 | 5862.64 | 1617.34 | 3.62 | 3.00E-04 |
| J47 | 5303.15 | 1516.70 | 3.50 | 5.00E-04 |
| J62 | 18642.74 | 8163.34 | 2.28 | 0.0224 |
| J69 | 25148.53 | 2407.72 | 10.45 | 0 |
| J81 | 31852.99 | 8163.34 | 3.90 | 1.00E-04 |
| J84 | 7275.44 | 1803.65 | 4.03 | 1.00E-04 |
| J94 | 9667.83 | 834.67 | 11.58 | 0 |
| J96 | 15962.32 | 1238.72 | 12.89 | 0 |
| J98 | 8444.45 | 371.27 | 22.74 | 0 |
| K21 | 3113.79 | 1096.91 | 2.84 | 0.0045 |
| K25 | 6758.43 | 1503.81 | 4.49 | 0 |
| K27 | 9550.85 | 1761.24 | 5.42 | 0 |
| K31 | 4172.31 | 1133.15 | 3.68 | 2.00E-04 |
| K40 | 17086.60 | 2461.81 | 6.94 | 0 |
| K43 | 15597.65 | 5772.46 | 2.70 | 0.0069 |
| K44 | 11394.05 | 3848.49 | 2.96 | 0.0031 |
| K51 | 28992.78 | 11544.59 | 2.51 | 0.012 |
| K52 | 1987.56 | 963.36 | 2.06 | 0.0391 |
| K56 | 15551.88 | 2721.53 | 5.71 | 0 |
| K59 | 3838.50 | 504.46 | 7.61 | 0 |
| K62 | 3838.36 | 1803.65 | 2.13 | 0.0333 |
| K63 | 4953.65 | 2144.35 | 2.31 | 0.0209 |
| K65 | 10620.45 | 2981.20 | 3.56 | 4.00E-04 |
| K66 | 16120.54 | 5772.46 | 2.79 | 0.0052 |
| K71 | 45667.81 | 4713.28 | 9.69 | 0 |
| K72 | 8298.69 | 830.37 | 9.99 | 0 |
| K76 | 2460.40 | 256.32 | 9.60 | 0 |
| K80 | 2803.83 | 357.98 | 7.83 | 0 |
| K82 | 1706.83 | 832.52 | 2.05 | 0.0403 |
| K92 | 12298.04 | 672.90 | 18.28 | 0 |
| L03 | 8620.18 | 3481.16 | 2.48 | 0.0133 |
| L23 | 4598.58 | 1455.34 | 3.16 | 0.0016 |
| L27 | 9896.05 | 4363.69 | 2.27 | 0.0233 |
| L28 | 8660.21 | 2886.56 | 3.00 | 0.0027 |
| L30 | 4448.80 | 1211.24 | 3.67 | 2.00E-04 |
| L70 | 16416.34 | 6665.40 | 2.46 | 0.0138 |
| L89 | 9881.85 | 1260.61 | 7.84 | 0 |
| M10 | 7117.62 | 774.72 | 9.19 | 0 |
| M17 | 2894.81 | 1155.55 | 2.51 | 0.0122 |
| M19 | 2617.54 | 1082.41 | 2.42 | 0.0156 |
| M23 | 7150.74 | 2721.53 | 2.63 | 0.0086 |
| M48 | 1220.62 | 331.17 | 3.69 | 2.00E-04 |
| M50 | 1631.73 | 649.34 | 2.51 | 0.012 |
| M62 | 59797.68 | 11544.59 | 5.18 | 0 |
| M80 | 4008.42 | 916.92 | 4.37 | 0 |
| M81 | 2106.59 | 624.47 | 3.37 | 7.00E-04 |
| N17 | 18219.73 | 2222.31 | 8.20 | 0 |
| N18 | 4165.59 | 581.59 | 7.16 | 0 |
| N19 | 7762.88 | 674.03 | 11.52 | 0 |
| N28 | 2172.90 | 392.41 | 5.54 | 0 |
| N31 | 12267.15 | 5163.09 | 2.38 | 0.0175 |
| N34 | 6335.88 | 1087.18 | 5.83 | 0 |
| N39 | 7231.94 | 740.77 | 9.76 | 0 |
| N40 | 2648.79 | 286.37 | 9.25 | 0 |
| N76 | 6609.86 | 2886.56 | 2.29 | 0.022 |
| N83 | 9403.96 | 3481.16 | 2.70 | 0.0069 |
| O10 | 22990.27 | 11544.59 | 1.99 | 0.0464 |
| O23 | 18793.21 | 6665.40 | 2.82 | 0.0048 |
| Q23 | 25806.45 | 11544.59 | 2.24 | 0.0254 |
| Q24 | 6881.65 | 2981.20 | 2.31 | 0.021 |
| R00 | 2446.47 | 931.64 | 2.63 | 0.0086 |
| R06 | 8769.07 | 4363.69 | 2.01 | 0.0445 |
| R16 | 4962.03 | 2041.42 | 2.43 | 0.0151 |
| R33 | 8343.69 | 1826.04 | 4.57 | 0 |
| R40 | 15908.37 | 4081.90 | 3.90 | 1.00E-04 |
| R47 | 13346.69 | 4363.69 | 3.06 | 0.0022 |
| R52 | 16117.33 | 5772.46 | 2.79 | 0.0052 |
| R56 | 93258.05 | 8163.34 | 11.42 | 0 |
| R57 | 25894.18 | 2800.40 | 9.25 | 0 |
| R63 | 32456.85 | 3651.03 | 8.89 | 0 |
| R64 | 21692.37 | 5163.09 | 4.20 | 0 |
| R65 | 35274.68 | 8163.34 | 4.32 | 0 |
| R73 | 4055.07 | 834.67 | 4.86 | 0 |
| R91 | 5961.50 | 1741.12 | 3.42 | 6.00E-04 |
| R94 | 5604.90 | 567.58 | 9.88 | 0 |
| S00 | 7134.48 | 2010.27 | 3.55 | 4.00E-04 |
| S04 | 15043.97 | 5772.46 | 2.61 | 0.0092 |
| S09 | 15507.76 | 5772.46 | 2.69 | 0.0072 |
| S22 | 5095.24 | 1649.98 | 3.09 | 0.002 |
| S42 | 6626.10 | 3085.80 | 2.15 | 0.0318 |
| S72 | 7836.70 | 1702.89 | 4.60 | 0 |
| S92 | 15053.90 | 5772.46 | 2.61 | 0.0091 |
| T86 | 15824.91 | 3848.49 | 4.11 | 0 |
| X48 | 55050.51 | 11544.59 | 4.77 | 0 |
| Z90 | 5816.48 | 2519.72 | 2.31 | 0.021 |
| Z95 | 7101.13 | 1741.12 | 4.08 | 0 |
| Z96 | 8086.46 | 3332.99 | 2.43 | 0.0153 |

**Table A6.** Regression analysis results of Model 6

(only present CCs with P-value < 0.05)

| ICD code of sixth CCs | Estimate | Std.. Error | t. value | P-value |
| --- | --- | --- | --- | --- |
| A04 | 15696.22 | 2093.82 | 7.50 | 0 |
| C11 | 11876.67 | 3455.44 | 3.44 | 6.00E-04 |
| C16 | 6728.06 | 2780.16 | 2.42 | 0.0155 |
| C48 | 17674.08 | 8101.07 | 2.18 | 0.0291 |
| D09 | 62612.78 | 11456.22 | 5.47 | 0 |
| D13 | 21298.00 | 8101.07 | 2.63 | 0.0086 |
| D32 | 4301.34 | 1673.86 | 2.57 | 0.0102 |
| D56 | 5916.52 | 1967.09 | 3.01 | 0.0026 |
| D61 | 6866.86 | 2959.49 | 2.32 | 0.0203 |
| D64 | 3488.01 | 547.65 | 6.37 | 0 |
| D76 | 44687.04 | 11456.22 | 3.90 | 1.00E-04 |
| E11 | 3794.30 | 168.50 | 22.52 | 0 |
| E14 | 3554.00 | 227.48 | 15.62 | 0 |
| E41 | 7288.30 | 1309.17 | 5.57 | 0 |
| E46 | 5678.36 | 1562.01 | 3.64 | 3.00E-04 |
| E78 | 1417.68 | 220.26 | 6.44 | 0 |
| E86 | 10541.81 | 5124.13 | 2.06 | 0.0397 |
| E87 | 1601.96 | 324.28 | 4.94 | 0 |
| F05 | 17734.68 | 3623.96 | 4.89 | 0 |
| G08 | 17804.02 | 8101.07 | 2.20 | 0.028 |
| G30 | 2492.35 | 869.04 | 2.87 | 0.0041 |
| G40 | 7705.65 | 626.25 | 12.30 | 0 |
| G41 | 17327.99 | 2959.49 | 5.86 | 0 |
| G80 | 49638.43 | 11456.22 | 4.33 | 0 |
| G91 | 5460.90 | 1253.75 | 4.36 | 0 |
| G93 | 6352.65 | 813.93 | 7.80 | 0 |
| H81 | -1718.84 | 572.13 | -3.00 | 0.0027 |
| I09 | 10049.02 | 817.95 | 12.29 | 0 |
| I11 | 1580.11 | 231.87 | 6.81 | 0 |
| I15 | 3895.12 | 1402.97 | 2.78 | 0.0055 |
| I21 | 12024.13 | 1353.62 | 8.88 | 0 |
| I42 | 5794.94 | 1317.71 | 4.40 | 0 |
| I44 | 5087.89 | 1185.62 | 4.29 | 0 |
| I49 | 3365.28 | 312.21 | 10.78 | 0 |
| I50 | 2176.50 | 379.16 | 5.74 | 0 |
| I61 | 9041.49 | 1149.75 | 7.86 | 0 |
| I62 | 7058.21 | 1576.62 | 4.48 | 0 |
| I63 | 3459.97 | 434.02 | 7.97 | 0 |
| I64 | 7054.40 | 1911.81 | 3.69 | 2.00E-04 |
| I65 | 5994.04 | 301.97 | 19.85 | 0 |
| I71 | 4877.66 | 2444.35 | 2.00 | 0.046 |
| I82 | 22926.10 | 2501.78 | 9.16 | 0 |
| J06 | -2051.98 | 686.78 | -2.99 | 0.0028 |
| J15 | 12341.91 | 690.37 | 17.88 | 0 |
| J45 | 3772.52 | 1173.29 | 3.22 | 0.0013 |
| J96 | 21474.63 | 661.10 | 32.48 | 0 |
| K13 | 9890.11 | 4677.84 | 2.11 | 0.0345 |
| K27 | 5042.87 | 1363.07 | 3.70 | 2.00E-04 |
| K36 | 14935.62 | 6614.74 | 2.26 | 0.024 |
| K56 | 4886.83 | 1996.60 | 2.45 | 0.0144 |
| K62 | 5511.66 | 2701.93 | 2.04 | 0.0414 |
| K92 | 11645.23 | 675.24 | 17.25 | 0 |
| L89 | 4545.93 | 1268.86 | 3.58 | 3.00E-04 |
| M10 | 2467.52 | 787.38 | 3.13 | 0.0017 |
| N41 | -5249.53 | 1860.95 | -2.82 | 0.0048 |
| R73 | 4116.57 | 841.21 | 4.89 | 0 |
| R90 | 3124.81 | 1149.75 | 2.72 | 0.0066 |
| R94 | 3743.48 | 744.39 | 5.03 | 0 |
| S00 | 4830.62 | 1639.47 | 2.95 | 0.0032 |
| S02 | 10617.91 | 3308.45 | 3.21 | 0.0013 |
| S36 | 17730.68 | 8101.07 | 2.19 | 0.0286 |
| S42 | 7333.56 | 3063.26 | 2.39 | 0.0167 |
| S52 | 8605.59 | 3063.26 | 2.81 | 0.005 |
| S72 | 4770.96 | 1363.07 | 3.50 | 5.00E-04 |
| T82 | 25348.57 | 4330.99 | 5.85 | 0 |
| Z49 | 55764.29 | 11456.22 | 4.87 | 0 |
| Z93 | 23087.27 | 3455.44 | 6.68 | 0 |
| Z95 | 7321.04 | 1382.58 | 5.30 | 0 |

**Table A7.** Regression analysis results of Model 7

(only present CCs with P-value < 0.05)

| ICD code of eighth CCs | Estimate | Std.. Error | t. value | P-value |
| --- | --- | --- | --- | --- |
| A04 | 22325.09 | 1518.65 | 14.70 | 0 |
| A16 | 18916.07 | 5780.85 | 3.27 | 0.0011 |
| A41 | 10620.54 | 3854.02 | 2.76 | 0.0059 |
| A49 | 5370.24 | 1743.44 | 3.08 | 0.0021 |
| B00 | 14283.12 | 4370.00 | 3.27 | 0.0011 |
| B35 | 7593.72 | 2985.43 | 2.54 | 0.011 |
| B37 | 34066.63 | 4720.10 | 7.22 | 0 |
| B49 | 32988.96 | 3656.27 | 9.02 | 0 |
| B94 | 30704.49 | 8175.25 | 3.76 | 2.00E-04 |
| C16 | 19513.32 | 5780.85 | 3.38 | 7.00E-04 |
| C20 | 11358.15 | 5780.85 | 1.96 | 0.0494 |
| C34 | 8631.27 | 3206.82 | 2.69 | 0.0071 |
| C50 | 12589.13 | 3486.14 | 3.61 | 3.00E-04 |
| C57 | 28745.31 | 11561.48 | 2.49 | 0.0129 |
| C67 | 14768.31 | 3854.02 | 3.83 | 1.00E-04 |
| C71 | 24217.93 | 8175.25 | 2.96 | 0.0031 |
| C79 | 17071.16 | 8175.25 | 2.09 | 0.0368 |
| D11 | 44404.37 | 11561.48 | 3.84 | 1.00E-04 |
| D32 | 5185.87 | 2523.24 | 2.06 | 0.0399 |
| D44 | 13056.50 | 5780.85 | 2.26 | 0.0239 |
| D46 | 72406.27 | 11561.48 | 6.26 | 0 |
| D50 | 4663.60 | 2225.38 | 2.10 | 0.0361 |
| D53 | 12049.85 | 4087.78 | 2.95 | 0.0032 |
| D56 | 16965.74 | 3854.02 | 4.40 | 0 |
| D61 | 10507.91 | 3854.02 | 2.73 | 0.0064 |
| D64 | 7773.56 | 518.18 | 15.00 | 0 |
| D68 | 17632.18 | 2013.01 | 8.76 | 0 |
| D69 | 4950.24 | 1014.85 | 4.88 | 0 |
| D70 | 2602.45 | 1269.70 | 2.05 | 0.0404 |
| D86 | 3394.11 | 1186.90 | 2.86 | 0.0042 |
| E03 | 3221.12 | 1145.50 | 2.81 | 0.0049 |
| E04 | 2029.75 | 688.50 | 2.95 | 0.0032 |
| E06 | 10295.47 | 3486.14 | 2.95 | 0.0031 |
| E11 | 7025.58 | 512.12 | 13.72 | 0 |
| E14 | 4230.52 | 747.43 | 5.66 | 0 |
| E23 | 30067.42 | 4087.78 | 7.36 | 0 |
| E27 | 6596.96 | 2985.43 | 2.21 | 0.0271 |
| E40 | 27194.18 | 11561.48 | 2.35 | 0.0187 |
| E41 | 20454.51 | 1480.86 | 13.81 | 0 |
| E43 | 18928.75 | 1293.27 | 14.64 | 0 |
| E44 | 10879.50 | 3337.75 | 3.26 | 0.0011 |
| E46 | 14249.36 | 1353.79 | 10.53 | 0 |
| E53 | 9993.67 | 2185.29 | 4.57 | 0 |
| E55 | 7586.26 | 3090.19 | 2.45 | 0.0141 |
| E72 | 5156.34 | 1056.22 | 4.88 | 0 |
| E77 | 11910.89 | 508.69 | 23.42 | 0 |
| E78 | 1789.67 | 319.47 | 5.60 | 0 |
| E79 | 2317.92 | 503.42 | 4.60 | 0 |
| E87 | 7132.75 | 299.03 | 23.85 | 0 |
| F01 | 4966.96 | 1784.44 | 2.78 | 0.0054 |
| F03 | 7323.70 | 1293.27 | 5.66 | 0 |
| F05 | 17555.99 | 5780.85 | 3.04 | 0.0024 |
| F06 | 9060.67 | 1954.67 | 4.64 | 0 |
| F20 | 5722.08 | 2652.69 | 2.16 | 0.031 |
| F32 | 7092.37 | 1186.90 | 5.98 | 0 |
| F41 | 3922.06 | 909.29 | 4.31 | 0 |
| F43 | 34237.30 | 11561.48 | 2.96 | 0.0031 |
| F45 | 7597.77 | 3486.14 | 2.18 | 0.0293 |
| F50 | 39562.61 | 5780.85 | 6.84 | 0 |
| G20 | 3757.78 | 1392.44 | 2.70 | 0.007 |
| G25 | 11822.74 | 3486.14 | 3.39 | 7.00E-04 |
| G31 | 2019.80 | 573.87 | 3.52 | 4.00E-04 |
| G37 | 7514.40 | 2013.01 | 3.73 | 2.00E-04 |
| G40 | 6491.76 | 1353.79 | 4.80 | 0 |
| G41 | 77054.88 | 8175.25 | 9.43 | 0 |
| G47 | 1680.17 | 579.55 | 2.90 | 0.0037 |
| G58 | 18315.22 | 4087.78 | 4.48 | 0 |
| G62 | 4900.56 | 2013.01 | 2.43 | 0.0149 |
| G91 | 6060.20 | 2111.22 | 2.87 | 0.0041 |
| G93 | 7839.05 | 1344.62 | 5.83 | 0 |
| H11 | 5211.64 | 2147.30 | 2.43 | 0.0152 |
| H25 | 5684.72 | 1927.34 | 2.95 | 0.0032 |
| H26 | 3316.14 | 1113.27 | 2.98 | 0.0029 |
| H34 | 33341.73 | 11561.48 | 2.88 | 0.0039 |
| H47 | 28707.54 | 6675.11 | 4.30 | 0 |
| H52 | 6248.77 | 2890.65 | 2.16 | 0.0306 |
| H59 | 18149.49 | 6675.11 | 2.72 | 0.0065 |
| H83 | 57691.78 | 11561.48 | 4.99 | 0 |
| I10 | 6299.15 | 405.85 | 15.52 | 0 |
| I11 | 5158.33 | 708.75 | 7.28 | 0 |
| I20 | 5240.33 | 1851.77 | 2.83 | 0.0047 |
| I21 | 15520.97 | 2985.43 | 5.20 | 0 |
| I24 | 13880.83 | 3656.27 | 3.80 | 1.00E-04 |
| I25 | 5394.86 | 539.47 | 10.00 | 0 |
| I27 | 2627.94 | 1199.57 | 2.19 | 0.0285 |
| I31 | 10952.93 | 1619.45 | 6.76 | 0 |
| I33 | 123688.54 | 11561.48 | 10.70 | 0 |
| I34 | 5517.95 | 2465.25 | 2.24 | 0.0252 |
| I35 | 4998.14 | 2360.32 | 2.12 | 0.0342 |
| I38 | 2445.36 | 1069.65 | 2.29 | 0.0222 |
| I46 | 86509.74 | 11561.48 | 7.48 | 0 |
| I47 | 7117.95 | 2985.43 | 2.38 | 0.0171 |
| I48 | 9708.72 | 826.85 | 11.74 | 0 |
| I49 | 5089.49 | 516.14 | 9.86 | 0 |
| I50 | 5031.20 | 600.86 | 8.37 | 0 |
| I61 | 13903.30 | 3656.27 | 3.80 | 1.00E-04 |
| I63 | 3636.78 | 726.61 | 5.01 | 0 |
| I64 | 10278.09 | 2890.65 | 3.56 | 4.00E-04 |
| I65 | 3211.92 | 427.01 | 7.52 | 0 |
| I66 | 4884.53 | 948.05 | 5.15 | 0 |
| I67 | 2651.59 | 432.25 | 6.13 | 0 |
| I69 | 5590.99 | 887.68 | 6.30 | 0 |
| I70 | 2667.43 | 284.34 | 9.38 | 0 |
| I71 | 6047.26 | 2411.07 | 2.51 | 0.0121 |
| I72 | 4455.10 | 2267.75 | 1.96 | 0.0495 |
| I74 | 6781.10 | 1927.34 | 3.52 | 4.00E-04 |
| I77 | 6933.76 | 1353.79 | 5.12 | 0 |
| I80 | 25083.32 | 1588.62 | 15.79 | 0 |
| I82 | 14257.65 | 2985.43 | 4.78 | 0 |
| I83 | 3853.13 | 1806.06 | 2.13 | 0.0329 |
| I84 | 4709.86 | 1293.27 | 3.64 | 3.00E-04 |
| I85 | 21329.07 | 6675.11 | 3.20 | 0.0014 |
| I95 | 7769.29 | 3656.27 | 2.12 | 0.0336 |
| J02 | 5629.03 | 2465.25 | 2.28 | 0.0224 |
| J06 | 1674.99 | 814.51 | 2.06 | 0.0397 |
| J15 | 14460.48 | 2225.38 | 6.50 | 0 |
| J18 | 9156.45 | 1199.57 | 7.63 | 0 |
| J22 | 23277.98 | 11561.48 | 2.01 | 0.0441 |
| J32 | 2095.20 | 954.47 | 2.20 | 0.0282 |
| J42 | 3141.49 | 857.99 | 3.66 | 3.00E-04 |
| J43 | 3184.64 | 912.10 | 3.49 | 5.00E-04 |
| J44 | 2938.03 | 581.72 | 5.05 | 0 |
| J63 | 59764.45 | 11561.48 | 5.17 | 0 |
| J66 | 56567.46 | 8175.25 | 6.92 | 0 |
| J69 | 16222.54 | 3854.02 | 4.21 | 0 |
| J84 | 6626.14 | 2411.07 | 2.75 | 0.006 |
| J93 | 105090.06 | 11561.48 | 9.09 | 0 |
| J94 | 10475.16 | 971.09 | 10.79 | 0 |
| J96 | 13786.16 | 1806.06 | 7.63 | 0 |
| J98 | 9057.83 | 604.92 | 14.97 | 0 |
| K06 | 34319.59 | 6675.11 | 5.14 | 0 |
| K11 | 61642.49 | 11561.48 | 5.33 | 0 |
| K21 | 3313.11 | 1573.85 | 2.11 | 0.0353 |
| K27 | 12767.83 | 1875.96 | 6.81 | 0 |
| K29 | 1116.70 | 392.14 | 2.85 | 0.0044 |
| K30 | 8902.24 | 3206.82 | 2.78 | 0.0055 |
| K31 | 3180.26 | 1505.73 | 2.11 | 0.0347 |
| K40 | 5466.65 | 2652.69 | 2.06 | 0.0393 |
| K52 | 2668.00 | 1145.50 | 2.33 | 0.0199 |
| K56 | 9730.79 | 2725.36 | 3.57 | 4.00E-04 |
| K59 | 3368.35 | 554.60 | 6.07 | 0 |
| K65 | 16300.15 | 5170.58 | 3.15 | 0.0016 |
| K72 | 16286.45 | 1134.44 | 14.36 | 0 |
| K74 | 15543.41 | 2465.25 | 6.31 | 0 |
| K76 | 2813.63 | 324.42 | 8.67 | 0 |
| K80 | 3126.72 | 425.88 | 7.34 | 0 |
| K82 | 3039.90 | 1108.15 | 2.74 | 0.0061 |
| K86 | 8113.93 | 3206.82 | 2.53 | 0.0114 |
| K92 | 10806.64 | 793.25 | 13.62 | 0 |
| L02 | 26317.44 | 11561.48 | 2.28 | 0.0228 |
| L08 | 5926.26 | 2890.65 | 2.05 | 0.0404 |
| L20 | 42208.68 | 11561.48 | 3.65 | 3.00E-04 |
| L23 | 4027.73 | 1603.81 | 2.51 | 0.012 |
| L29 | 6091.55 | 2890.65 | 2.11 | 0.0351 |
| L30 | 7748.21 | 1505.73 | 5.15 | 0 |
| L50 | 8161.91 | 2804.36 | 2.91 | 0.0036 |
| L89 | 9386.58 | 1193.18 | 7.87 | 0 |
| M10 | 7365.28 | 885.09 | 8.32 | 0 |
| M19 | 3081.97 | 1353.79 | 2.28 | 0.0228 |
| M23 | 21519.90 | 4087.78 | 5.26 | 0 |
| M35 | 12050.89 | 4370.00 | 2.76 | 0.0058 |
| M48 | 2353.25 | 474.19 | 4.96 | 0 |
| M50 | 2181.13 | 1003.35 | 2.17 | 0.0297 |
| M80 | 5201.97 | 1353.79 | 3.84 | 1.00E-04 |
| M81 | 3982.72 | 752.13 | 5.30 | 0 |
| M87 | 5316.40 | 2652.69 | 2.00 | 0.0451 |
| N13 | 6452.78 | 1705.14 | 3.78 | 2.00E-04 |
| N17 | 24392.29 | 3854.02 | 6.33 | 0 |
| N18 | 6302.49 | 796.99 | 7.91 | 0 |
| N19 | 4821.13 | 887.68 | 5.43 | 0 |
| N20 | 1555.40 | 625.63 | 2.49 | 0.0129 |
| N21 | 9339.55 | 3206.82 | 2.91 | 0.0036 |
| N28 | 2014.13 | 415.50 | 4.85 | 0 |
| N31 | 12553.58 | 4720.10 | 2.66 | 0.0078 |
| N32 | 4165.09 | 2076.90 | 2.01 | 0.0449 |
| N34 | 8978.54 | 1285.27 | 6.99 | 0 |
| N36 | 16069.29 | 4720.10 | 3.40 | 7.00E-04 |
| N39 | 10485.59 | 857.99 | 12.22 | 0 |
| N40 | 3246.83 | 344.91 | 9.41 | 0 |
| N42 | 8010.83 | 2890.65 | 2.77 | 0.0056 |
| N45 | 15141.94 | 6675.11 | 2.27 | 0.0233 |
| N94 | 8061.17 | 3656.27 | 2.20 | 0.0275 |
| O99 | 19148.58 | 5780.85 | 3.31 | 9.00E-04 |
| Q21 | 14327.38 | 6675.11 | 2.15 | 0.0318 |
| R04 | 8674.76 | 4087.78 | 2.12 | 0.0338 |
| R33 | 7461.43 | 1743.44 | 4.28 | 0 |
| R41 | 23898.43 | 5170.58 | 4.62 | 0 |
| R55 | 9032.56 | 4370.00 | 2.07 | 0.0387 |
| R57 | 39457.02 | 3656.27 | 10.79 | 0 |
| R63 | 29157.78 | 3206.82 | 9.09 | 0 |
| R64 | 21181.11 | 5170.58 | 4.10 | 0 |
| R73 | 3974.48 | 1139.93 | 3.49 | 5.00E-04 |
| R77 | 35260.49 | 5780.85 | 6.10 | 0 |
| R82 | 16890.76 | 8175.25 | 2.07 | 0.0388 |
| R91 | 5413.19 | 2312.65 | 2.34 | 0.0192 |
| R94 | 6814.72 | 698.40 | 9.76 | 0 |
| S00 | 7305.55 | 3337.75 | 2.19 | 0.0286 |
| S14 | 107857.51 | 11561.48 | 9.33 | 0 |
| S37 | 17497.83 | 4370.00 | 4.00 | 1.00E-04 |
| S42 | 10054.32 | 3656.27 | 2.75 | 0.006 |
| S53 | 57810.50 | 11561.48 | 5.00 | 0 |
| S83 | 14800.12 | 5780.85 | 2.56 | 0.0105 |
| T00 | 10811.72 | 5170.58 | 2.09 | 0.0365 |
| T30 | 18429.10 | 4720.10 | 3.90 | 1.00E-04 |
| T79 | 31050.60 | 11561.48 | 2.69 | 0.0072 |
| T86 | 18774.49 | 5780.85 | 3.25 | 0.0012 |
| T90 | 70375.94 | 8175.25 | 8.61 | 0 |
| T95 | 25037.54 | 11561.48 | 2.17 | 0.0303 |
| Z86 | 4627.60 | 2312.65 | 2.00 | 0.0454 |
| Z90 | 9542.63 | 3090.19 | 3.09 | 0.002 |
| Z95 | 6668.86 | 2185.29 | 3.05 | 0.0023 |

**Table A8.** Summary of CCs levels after optimization

| Level | | ICD code of disease |
| --- | --- | --- |
| MCC | G97, T06, B37, F43, A40, D46, B95, R23, M88, R65, Z49, I33, D76, I46, K75, M84, D89, Y42, C91, R02, G08, F00, A04, K36, K35, J18, J15, K92, D45, K71, E86, J94, I09, K38, D07, J62, E23, G04, R13, I66, J98, E77, F06, E10, D61, C16, H35, L28, L27, K65, I72, I26, M32, I42, E70, M05, Z96, A18, I31, E15, J46, D37, K72, I44, I35, K27, K56, I71, R46, I08, D75, E44, J85, M24, G25, G81, N39, K85, L89, K58, E74, G12, J39, F42, S30, D35, F45, B35, M46, F03, D15, R53, Z95, J84, N12, L29, L08, F20, I88, L03, E72, Q60, G03, K57, C11, B02, H18, A09, M81, F41, H36, I83, A71, G44, M60, M06, N26, E03, J43, J42, K82, J44, M19, H91, T91, H90, J02, K10, H93, G70, I24, R17, M91, K52, M11, D48, X12, D53, H92, H72, E75, J31, E22, M89, K29, T92, D81, T84, S63, Y57, Q17, J20, J40, G95, G23, R56, H81, M41, G57, Z51, Q80, E65, N02, F95, D52, C41, G82, K50, K07, H55, I36, R11, B43, H83, G96, M62, X48, J04, I60, K51, J13, E01, H04, K61, F29, J93, T90, S53, J66, R77, H34, T79, B94, E40, T95, C71, J22, H59, S37, C79, K74, S83, C67, Q21, C50, C20, T00, R55, K30, R04, N94, N42, N13, H25, M87, N32 | |
| mCC | | J95, B44, Q12, I12, B49, S73, A41, I85, J12, T82, Z43, H47, S82, E84, D27, F01, H52, N34, G36, I87, I15, C22, N36, N19, E14, D64, I97, R09, I70, E06, G53, I38, B00, R91, M33, K26, I10, D41, M95, K22, G56, S22, T86, R18, G72, D72, F50, G20, D47, I25, E53, I11, K20, E78, B24, Q24, L30, I27, A53, R19, E55, S05, A16, I13, K55, H44, Z03, G51, D34, I51, Z86, G71, S27, E83, N49, E79, J47, M50, N86, H17, G31, K83, R31, G45, K02, N20, K13, K05, Q76, L12, M86, G63, J34, M51, Q61, H31, N41, J37, B99, S50, Z08, E02, D01, M70, R32, J35, R51, R39, R52, E89, T10, J81, R42, E26, D40, D02, K91, K60, K28, T85, Q23, F31, O10, R64, O23, J38, N17, E41, K40, L70, K66, R40, K43, S09, S04, G90, D68, N31, G40, I95, I05, D56, D38, F09, N83, H02, R33, S72, G91, D50, C34, S00, M10, I77, K25, S42, N76, F32, E07, J45, Z90, R94, H11, G62, G93, B16, I84, G37, R16, K63, I47, L23, G30, I69, D18, N18, R73, K62, E05, A49 |
| non-CC | | Z98, L88, G92, C25, D73, R57, D84, J69, J96, E16, H46, H70, D69, D29, D33, M15, D44, M76, K14, I34, N62, R00, R25, H66, K11, H54, G50, E24, F93, D70, G47, K21, B19, N60, E80, J32, I40, I86, K03, G61, T93, G06, K81, J11, H60, J00, E12, H65, J33, E51, J06, J03, M90, G43, J01, T88, M75, I00, J30, B34, F51, R50, K04, M00, M94, R30, R54, T07, K06, O11, L20, R22, I01, O99, N23, M72, H61, B30, T94, R49, E71, G41, J63, C57, L02, R41, M23, E43, T30, F05, E46, M35, A81, I80, R63, I61, C23, I82, G52, S10, E34, E66, F07, N99, N61, I48, I74, R47, I21, K73, D11, N25, M98, E21, B09, N30, E00, D55, S01, K86, G21, T81, L72, E11, L50, D36, I49, R06, M80, E87, E85, D86, K44, M82, S92, E13, E27, I28, J92, K59, I50, G58, E63, K76, A00, K31, I45, D17, N40, R21, M12, K80, L51, O34, Z85, R03, R80, M17, I65, I20, I67, E04, H26, N28, M48, D09, G80, Z93, D13, S36, C48, Z94, S14, S02, R14, S52, Z54, S86, I62, I64, R35, C15, N85, D43, D32, R68, N90, Z87, I63, H33, B18, L40, R90, Q44, R82, D24, H49, I99, A51, C76, E88, R10, I73, L97, L98, D00, F30, N21, S39, H53, B17, L82, T09, L25, N63, F79, H73, C95, M53, T13, H28, B65, T25, T11, H43, C21, N45, H01, B36, R20, D51, L57, B15, L80, Q00, N95, Z47, S80, H00, D14, H27, H30, X32, B40 |
